# Mucoactive agents in bronchiectasis: a systematic review and meta-analysis

**DOI:** 10.1183/16000617.0014-2026

**Published:** 2026-06-24

**Authors:** Benjamin McCullough, John Busby, Brenda O'Neill, Bronwen Connolly, Rebecca H. McLeese, Dermot Linden, Daniel F. McAuley, J. Stuart Elborn, Anthony De Soyza, James D. Chalmers, Mike Clarke, Judy M. Bradley

**Affiliations:** 1 Wellcome Trust–Wolfson Northern Ireland Clinical Research Facility, Queen's University Belfast, Belfast, UK; 2 Centre for Public Health, Queen's University Belfast, Belfast, UK; 3 Institute of Nursing and Health Research, Ulster University, Belfast, UK; 4 Wellcome–Wolfson Institute for Experimental Medicine, Queen's University Belfast, Belfast, UK; 5 Department of Physiotherapy, The University of Melbourne, Melbourne, Australia; 6 Population Health Sciences Institute, Faculty of Medical Sciences, Newcastle University, Newcastle, UK; 7 Scottish Centre for Respiratory Research, University of Dundee, Ninewells Hospital and Medical School, Dundee, UK

## Abstract

**Background:**

Mucoactive agents aim to improve mucociliary clearance in bronchiectasis, disrupting the cycle of impaired clearance, infection and inflammation. Registry data show 28% of patients use these agents, but prescribing varies due to limited evidence. We aimed to update evidence on mucoactive agents’ effects in adults with bronchiectasis.

**Methods:**

We conducted a systematic review and meta-analysis, searching Medline, Embase, CENTRAL and trial registries to 1 October 2025. Eligible studies included adults with bronchiectasis, evaluating any mucoactive agent *versus* placebo or control. Cystic fibrosis and paediatric studies were excluded. The primary outcome was exacerbation frequency; secondary outcomes included lung function, quality of life and adverse events.

**Findings:**

From 1512 records, 24 studies (20 randomised, four observational: n=7051) evaluated eight mucoactive agents (ambroxol, bromhexine, carbocisteine, erdosteine, hypertonic saline, mannitol, N-acetylcysteine, rhDNase). Most trials had high or some risk of bias. Seven randomised studies (n=1259) reported exacerbations; pooled analysis did not demonstrate a statistically significant difference in annualised exacerbation incidence (mean difference −0.40 per patient per year, 95% CI –1.04–0.24; p=0.22; I^2^=91%; very low certainty). Nine studies (n=767) reported percent predicted forced expiratory volume in 1 s (FEV_1_ % pred), showing a small mean increase of 3.23% (95% CI 0.31–6.15; p=0.03; I^2^=69.9%; very low certainty). Pooled analyses did not demonstrate significant differences for other spirometry measures, quality of life or adverse events.

**Interpretation:**

Pooled evidence did not show a reduction in exacerbations and FEV_1_ % pred improvements were small. Evidence certainty was low/very low, meaning overall clinical benefit remains uncertain, highlighting the need for targeted trials of specific agents and subgroups.

## Shareable abstract

**Mucoactive agents were not associated with significant reductions in exacerbations or improved quality of life in bronchiectasis. Evidence certainty was very low, highlighting further trials are needed to identify which patients, if any, may benefit.** https://bit.ly/4msQS5G

## Introduction

Bronchiectasis is characterised by permanent dilatation of the bronchi, presenting as a clinical syndrome of chronic cough, mucus hypersecretion and recurrent respiratory exacerbations [1]. Exacerbations, defined as a sustained worsening of symptoms including increased sputum volume for 48 h or more, requiring additional treatment, significantly contribute to morbidity and disease progression [2]. Bronchiectasis imposes a substantial clinical and socioeconomic burden on patients and healthcare systems, making the identification of treatments to prevent exacerbations a top research priority [3, 4].

The self-perpetuating “vicious vortex” of airway dysfunction, infection, inflammatory response and structural disease is well described in bronchiectasis [5]. Increased sputum viscoelasticity correlates with lung function impairment, radiological disease severity and systemic inflammation [6]. Airway clearance management, including airway clearance techniques (ACTs), devices and mucoactive agents, aims to break this cycle by enhancing secretion clearance, thereby reducing mucus retention, inflammation, infection and ultimately exacerbations.

Currently, airway clearance management is primarily used in patients with severe bronchiectasis, reflecting a reactive treatment approach [7]. Despite international guidelines emphasising its importance, implementation remains inconsistent; for example, only 52.2% of European patients regularly use ACTs [7, 8]. Use of mucoactive agents as part of airway clearance is even lower, with only 28% of bronchiectasis patients employing these treatments, predominantly those with severe disease [7]. Mucoactive agents aim to improve mucus clearance and reduce hypersecretion through various mechanisms (table S1). Expectorants such as hypertonic saline (HTS) hydrate mucus and induce cough, mucoregulators such as carbocisteine control secretion, mucolytics such as N-acetylcysteine decrease mucus viscosity, and mucokinetics such as surfactants enhance mucociliary movement [9, 10]. While geographic variability and limited access contribute to this low uptake, insufficient evidence of benefit likely explains the limited use [7].

Guideline recommendations on mucoactive agents vary, reflecting uncertainty. The 2017 European Respiratory Society (ERS) guidelines recommended long-term use of mucoactive agents for symptom management if ACT fails, while the British Thoracic Society suggests trials for patients with expectoration difficulty irrespective of ACT [11, 12]. In contrast, the Thoracic Society of Australia and New Zealand and Spanish Society of Pulmonology and Thoracic Surgery do not recommend routine use due to insufficient evidence [13, 14]. The recently updated 2025 ERS guidelines maintain a conditional recommendation for mucoactive agents, but are more cautious, with evidence certainty downgraded from “low” to “very low” to reflect persistent uncertainty about their effectiveness [15]. The review included in the 2025 guideline contains nine randomised trials but did not incorporate observational data or recent trial results, suggesting that a comprehensive, updated evidence synthesis could further inform this area of ongoing uncertainty. While randomised trials remain the gold standard for assessing mucoactive agent efficacy, observational studies may complement trial evidence by providing insight into real-world prescribing patterns and outcomes in large bronchiectasis patient populations not fully captured in randomised designs [16].

Patients have emphasised the need for robust evaluation of mucoactive agents [3]. If effective, approximately three-quarters of patients with bronchiectasis may be missing an affordable therapy that targets key symptoms and reduces exacerbations. Conversely, if ineffective, the one-quarter currently using them may be receiving unnecessary therapy [7, 9]. Patient-identified treatment burdens include nebuliser device availability, cost and cleaning requirements [15].

Conflicting findings from individual studies underscore the urgent need for research to determine optimal dosages, durations and combinations of mucoactive agents, with systematic reviews essential for addressing inconsistencies [10, 17, 18]. The most recent Cochrane review (searches through June 2013) found limited effectiveness for mucoactive agents in bronchiectasis, based on low-quality evidence from four trials (n=528) [19]. However, data could not be meta-analysed as results were reported in a format that could not be aggregated and HTS was excluded as it was not prespecified as a mucolytic agent. HTS was evaluated in a separate 2014 Cochrane review of inhaled hyperosmolar agents for bronchiectasis, but limited data prevented clear conclusions regarding effectiveness [20]. A 2017 systematic review of inhaled mucoactive agents for non-cystic fibrosis (CF) chronic lung diseases, including bronchiectasis, found mannitol prolonged time to first exacerbation but did not reduce exacerbation rates, while HTS reduced exacerbations at 3 months but not at 12 months [21]. However, the review was limited by small, short duration trials that left long-term effectiveness uncertain. Oral mucoactive agents were not reviewed.

Since these reviews, several large studies have been published, including the CLEAR trial of HTS 6% and carbocisteine conducted by our group, making an updated evidence synthesis both timely and necessary [22]. Our review evaluates all mucoactive categories (mucolytics, expectorants, mucoregulators and mucokinetics), providing a comprehensive assessment of available therapeutic options.

Therefore, this systematic review and meta-analysis aims to update the evidence for mucoactive treatment in adults with bronchiectasis, incorporating recent randomised trials and observational cohort studies.

## Methods

### Search strategy and selection criteria

The methods for this systematic review and meta-analysis are detailed in a protocol registered with the International Prospective Register of Systematic Reviews (PROSPERO: CRD42024545050) [23]. Results are reported in accordance with the Preferred Reporting Items for Systematic Reviews and Meta-Analyses (PRISMA) guidelines [24].

Randomised trials and observational cohort studies with a comparator group that evaluated at least one mucoactive agent *versus* standard care, placebo or a control group, in adults (≥16 years of age) were eligible. Paediatric studies and those involving patients with CF were excluded. Mucoactive agents were defined as any pharmacological treatment aimed at promoting mucus clearance from the lungs.

Electronic databases systematically searched for relevant studies included Medline, Embase, Cochrane Central Register of Controlled Trials (CENTRAL), EU Clinical Trial Register, ClinicalTrials.gov and the World Health Organization (WHO) trial registry portal. The search covered records from inception to 1 October 2025. No language restrictions were applied in the search, but only studies published in English were included in the review.

Reference lists from included studies and relevant reviews were manually screened to identify any further eligible studies. Our analysis also incorporated all included studies from the Cochrane review [19].

The search strategy employed a combination of key terms for bronchiectasis, mucoactive agents and study designs (table S2). Search results were imported into Covidence software (Veritas Health Innovation, Melbourne, Australia) for screening, data extraction and risk of bias assessment. Duplicate reports were automatically detected and excluded.

All authors participated in the screening process, with at least two authors independently screening the identified reports by title and abstract against the inclusion criteria. They then reviewed the full text of potentially relevant studies for eligibility. Multiple reports of the same study (*e.g.* conference abstracts, journal articles, *post hoc* analyses) were considered as a single study, with the full-text article cited here. Abstracts were included if they provided adequate data on relevant outcomes. Disagreements on study inclusion were resolved by consensus among the authors.

### Data analysis

Two authors from the review team independently extracted study characteristics and outcome data from included studies in duplicate and completed a confirmed consensus data extraction template on Covidence. Study characteristics included study design, study duration, diagnostic criteria for bronchiectasis, sample size, participant characteristics, number of withdrawals, reasons for withdrawal, challenge test conducted and concomitant therapies (tables S3 and S4). Outcome measures assessed aligned with a core outcome set for bronchiectasis, including pulmonary exacerbations, lung function, shortness of breath, cough, exercise tolerance, sputum characteristics, quality of life, patient perception of health, admissions to hospital and adverse events (AEs) [25]. Inclusion criteria were also collated to assess consistency across trials (table S5).

AEs were defined as any undesirable experiences or symptoms occurring during medical treatment or study participation. These included physiological discomfort (*e.g.* chest discomfort), infections (*e.g.* lower respiratory tract infection), respiratory issues (*e.g.* cough, dyspnoea) and systemic reactions (*e.g.* headache, nausea).

Two authors from the review team independently assessed the risk of bias of included studies in duplicate and completed a confirmed consensus quality assessment template on Covidence. The revised Cochrane Risk-of-Bias (RoB 2) tool was used for randomised trials [26] and the Risk-Of-Bias In Non-randomised Studies of Interventions (ROBINS-I) tool was used for nonrandomised studies [27]. Risk of bias assessments were visualised using the Risk-of-bias VISualization (robvis) R package and web application [28]. Results from randomised trials and observational studies were presented separately, with subgroup analysis used in instances where both study types contributed to the same outcome to ensure clear distinction. As several review authors contributed to the CLEAR trial [22], data extraction and risk-of-bias assessments for that study were performed independently by authors not involved in its conduct, in line with Cochrane guidance on managing competing interests [29].

For binary variables, data were calculated as odds ratios with 95% confidence intervals. Data for continuous variables were calculated as mean differences (MDs) with 95% confidence intervals.

Where clinically appropriate, multiple treatment arms involving mucoactive agents within individual studies were combined into a single group when they represented different doses or closely related formulations of the same intervention with shared clinical intent and a common comparator. This approach was used to maintain appropriate pairwise comparisons and to avoid unit-of-analysis errors arising from double counting participants in shared comparator groups, which can artificially inflate precision and study weighting. Similarly, multiple comparator or control arms within individual studies (*e.g.* no treatment and standard care) were combined into a single “no mucoactives” group for meta-analysis.

Meta-analyses were conducted using DerSimonian–Laird random-effects models [30]. The I^2^ statistic was used to quantify the variability in effect estimates attributable to heterogeneity rather than to chance [31]. In general, I^2^ values of 25%, 50% and 75% indicate low, moderate and substantial heterogeneity, respectively, but interpretation may vary based on context [32]. Prediction intervals were calculated to estimate the expected range of effect in a future study while accounting for between-study heterogeneity. Data analysis was performed using STATA (version 18) and R package Meta [33] (version 8.2-1). Studies were classified as outliers when their 95% confidence interval showed no overlap with the 95% confidence intervals of the pooled effect. *Post hoc* sensitivity analyses of the primary outcome were conducted to explore potential sources of heterogeneity related to trial inclusion criteria and comparator type.

Studies with factorial designs evaluating multiple mucoactive interventions required special analytical consideration to prevent unit-of-analysis errors from shared control groups. In this case, separate meta-analyses were conducted for each mucoactive agent, with primary analyses followed by sensitivity analyses using the alternative agent from the same factorial study to ensure robust interpretation [34]. Study authors were contacted to obtain individual treatment-arm data to allow appropriate disaggregation of results. For crossover trials, paired within-participant data were rarely reported; therefore, end-point summary statistics for intervention and comparator periods were analysed as parallel-group comparisons [34].

The quality of evidence was evaluated using the Grading of Recommendations, Assessment, Development and Evaluation (GRADE) framework [35]. Study design, risk of bias, inconsistency, indirectness, imprecision and publication bias were evaluated and evidence profiles and summary of findings tables for each outcome were generated using the GRADEpro Guideline Development Tool (www.gradepro.org). For outcomes with more than 10 studies, publication bias was assessed by examining funnel plots for each outcome and Egger's test of asymmetry [36] (figures S13–S14).

### Role of funding source

There was no direct funding for this study.

## Results

**FIGURE 1:**
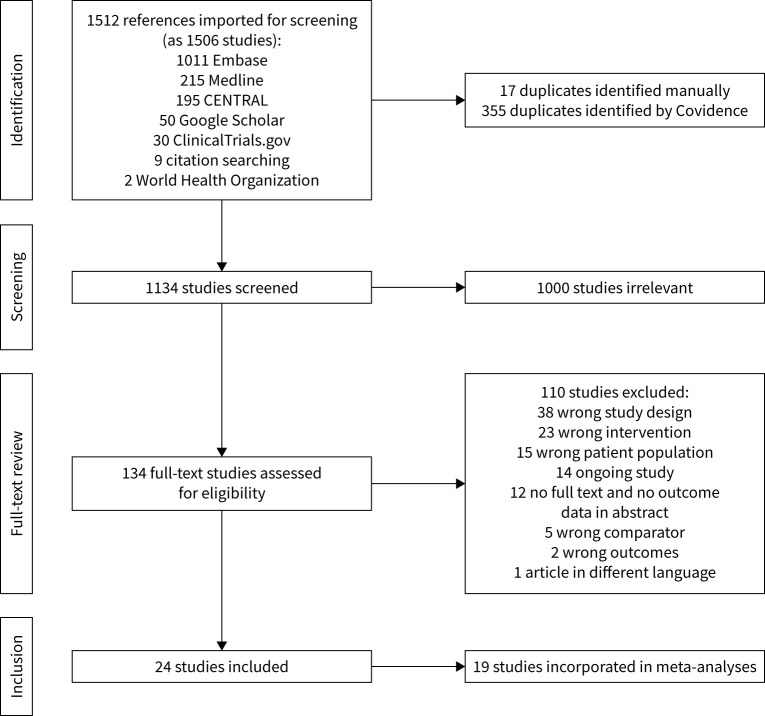
Study selection. CENTRAL: Cochrane Central Register of Controlled Trials.

A total of 1512 records were identified through database searches and citation searching. After removing duplicates, 1134 titles and abstracts were screened, of which 1000 were excluded as they did not meet eligibility criteria. Of the 134 full-text articles assessed for eligibility, 110 were excluded for reasons such as ineligible study design, intervention or population (table S5), and 14 were ongoing studies (table S6). Ultimately, 24 studies met inclusion criteria, including 20 randomised controlled trials (RCTs) and four nonrandomised observational studies (figure 1). These evaluated eight mucoactive agents (HTS, mannitol, erdosteine, N-acetylcysteine, carbocisteine, rhDNase, bromhexine and ambroxol hydrochloride) across 7051 patients. Characteristics of included studies are reported in table S3.

Among the 20 randomised trials, 11 were judged to have high risk of bias [37–47], eight had some concerns [10, 48–53] and one was rated as low risk [17] (figure S1). For the four nonrandomised studies, two had serious risk of bias concerns [54, 55], one had moderate concerns [56] and one could not be assessed due to insufficient information [57].

**FIGURE 2:**
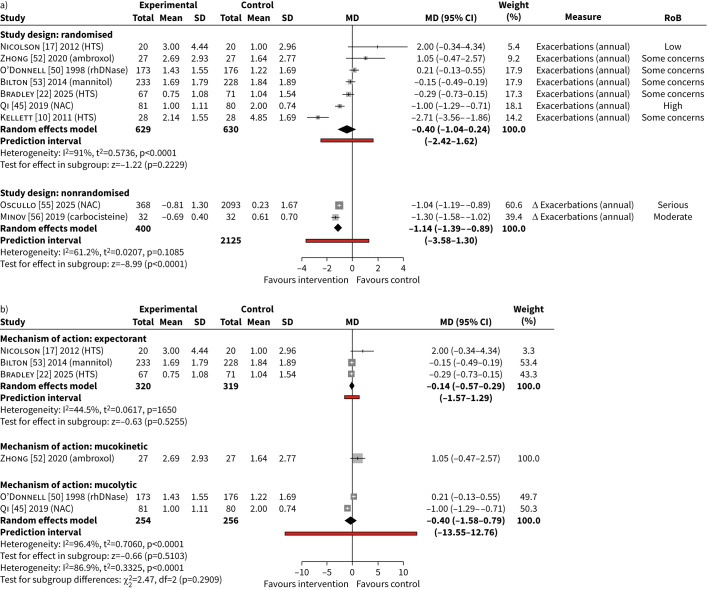
Meta-analysis of annualised exacerbation incidence between mucoactive treatment and control groups. a) Mean differences (MDs) and changes in exacerbations per year from nine studies were analysed. b) Randomised studies, minus outlier Kellett *et al.* [10], grouped by mucoactive mechanism of action. Red lines indicate prediction interval, reflecting expected range of effects in a new study, accounting for between-study differences. Studies are listed as first author, reference, publication year. HTS: hypertonic saline; NAC: N-acetylcysteine; RoB: overall risk of bias judgement.

The primary outcome of annualised exacerbation incidence showed substantial heterogeneity (I^2^=92.5%, p<0.0001) in the overall meta-analysis of nine studies (seven randomised trials and two observational cohort studies; 3729 patients) (figure 2a). Subgroup analysis by study design showed a significant difference between randomised and nonrandomised studies (test for subgroup differences: p=0.04). In randomised studies, pooled analysis found mucoactive treatment was not associated with a statistically significant difference in annualised exacerbation incidence compared with control (MD −0.40, 95% CI –1.04–0.24; p=0.22; prediction interval –2.42–1.62), with substantial heterogeneity (I^2^=91%, p<0.0001). In nonrandomised studies, mucoactive treatment was associated with a mean decrease of 1.14 exacerbations per year (95% CI –1.39––0.89; p<0.0001; prediction interval –3.58–1.30), with moderate-to-substantial heterogeneity (I^2^=61.2%, p=0.11).

Removal of an identified outlier study (Kellett *et al*. [10]) from the randomised subgroup did not substantially change the mean effect or reduce heterogeneity, with the MD showing a decrease of 0.09 exacerbations per year (95% CI –0.64–0.46; p=0.75; prediction interval 1.74–1.56), with substantial heterogeneity (I^2^=86.9%, p<0.0001). A sensitivity analysis excluding high risk of bias randomised studies (Qi *et al.* [45]) showed the MD as a decrease of 0.15 exacerbations per year (95% CI –1.31–1.02; p=0.80; prediction interval –3.95–3.65), with substantial heterogeneity (I^2^=88.9%, p<0.0001). A further sensitivity analysis including carbocisteine data from Bradley *et al.* [22] (MD −0.11, 95% CI −0.53–0.31) instead of the trial's HTS data showed the MD as a decrease of 0.05 exacerbations per year (95% CI –0.61–0.50; p=0.85; prediction interval –1.71–.60), with substantial heterogeneity (I^2^=87.2%, p<0.0001).

In a *post hoc* sensitivity analysis restricted to exacerbation-enriched populations (≥2 exacerbations in the preceding year) (table S4), mucoactive therapy was not associated with a reduction in annualised exacerbation incidence (three studies: Nicolson *et al.* [45], Bilton *et al.* [53] and Qi *et al.* [45]; MD −0.12, 95% CI −1.41–1.17; p=0.85), with substantial heterogeneity remaining (I^2^=89.4%, p<0.0001). Similarly, a *post hoc* sensitivity analysis restricted to RCTs enrolling patients with chronic sputum production or difficulty expectorating sputum (table S4), mucoactive therapy was not associated with a reduction in annualised exacerbation incidence (five studies: Nicolson *et al.* [45], Zhong *et al.* [52], O’Donnell *et al.* [50], Bilton *et al.* [53] and Bradley *et al.* [22]; MD 0.02, 95% CI −0.29–0.32; p=0.92), with moderate heterogeneity observed (I^2^=53.0%, p=0.07). In a further *post hoc* sensitivity analysis excluding RCTs using potentially active comparators (isotonic saline or low-dose mannitol), mucoactive therapy was not associated with a reduction in annualised exacerbation incidence (three studies: O’Donnell *et al.* [50], Bradley *et al.* [22] and Qi *et al.* [45]; MD −0.37, 95% CI −1.07–0.34; p=0.31), with substantial heterogeneity remaining (I^2^=93.0%, p<0.0001).

Subgroup analysis of randomised studies by mucoactive mechanism of action showed that for expectorants (n=3), the MD was a decrease of 0.14 exacerbations per year (95% CI –0.57–0.29; p=0.53; prediction interval 1.57–1.29) (figure 2b), with moderate heterogeneity (I^2^=44.5%, p=0.17).

**FIGURE 3:**
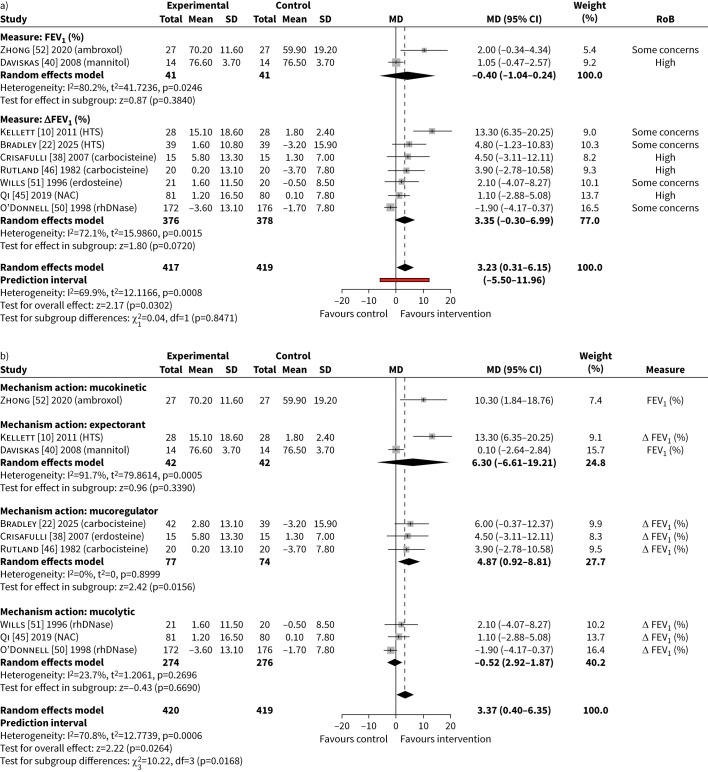
Meta-analysis of mean differences (MDs) in forced expiratory volume in 1 s (FEV_1_) percentage predicted between mucoactive treatment and control groups. Mean differences and changes in FEV_1_% from nine randomised studies were analysed. Red line indicates prediction interval, reflecting expected range of effects in a new study, accounting for between-study differences. b) Studies grouped by mucoactive mechanism of action, with carbocisteine data from Bradley *et al.* [22]. Studies are listed as first author, reference, publication year. HTS: hypertonic saline; NAC: N-acetylcysteine; RoB: overall risk of bias judgement.

For secondary outcomes such as lung function, meta-analysis of percent predicted forced expiratory volume in 1 s (FEV_1_ % pred) and ΔFEV_1_ % pred data from nine randomised trials (767 patients) showed that mucoactive treatment was associated with improved FEV_1_ % pred. The overall MD was +3.23% (95% CI 0.31–6.15; p=0.03; prediction interval –5.50–11.96; figure 3a), with substantial heterogeneity (I^2^=69.9%, p=0.0008). A sensitivity analysis including carbocisteine data from Bradley *et al.* [22] (MD −6.00, 95% CI −0.37–12.37) instead of the trial's HTS data showed the overall MD was +3.37% (95% CI 0.40–6.35; p=0.03; prediction interval –5.58–12.33), also with substantial heterogeneity (I^2^=70.8%, p=0.0006).

Subgroup analysis of randomised studies by mucoactive mechanism of action showed that for mucoregulators (n=3), the overall MD was +4.87% (95% CI 0.92–8.81; p=0.02; prediction interval –3.79–13.52) (figure 3b), with no heterogeneity (I^2^=0%, p=0.90).

Meta-analysis of 11 randomised trials (1277 patients) showed that mucoactive treatment was not associated with differences in FEV_1_ (L) or ΔFEV_1_ (L) (figure S3). The overall MD was 0.01 L (95% CI –0.08–0.09; p=0.86) but with heterogeneity that was close to the threshold for “substantial” (I^2^=72.8%). A sensitivity analysis including carbocisteine data from Bradley *et al.* [22] instead of the trial's HTS data showed the overall MD was 0.01 L (95% CI –1.08–0.10; p=0.83), also with substantial heterogeneity.

Meta-analysis of ten randomised trials (1210 patients) showed that mucoactive treatment was not associated with differences in forced vital capacity (FVC) (L) or ΔFVC (L) (figure S4). The MD was 0.01 L (95% CI –0.02–0.05; p=0.45). No statistical heterogeneity was observed (I^2^=0.0%), suggesting consistent findings across the trials. A sensitivity analysis including carbocisteine data from Bradley *et al.* [22] instead of the trial's HTS data showed the overall MD was 0.01 L (95% CI −0.02–0.05; p=0.47; I^2^=0.0%).

Meta-analysis of four randomised trials (447 patients) showed that mucoactive treatment was not associated with differences in FVC percentage of predicted (FVC%) or ΔFVC% (figure S5). The MD was 2.8% (95% CI –4.8–10.3; p=0.47) but substantial heterogeneity was observed (I^2^=95.4%).

**FIGURE 4:**
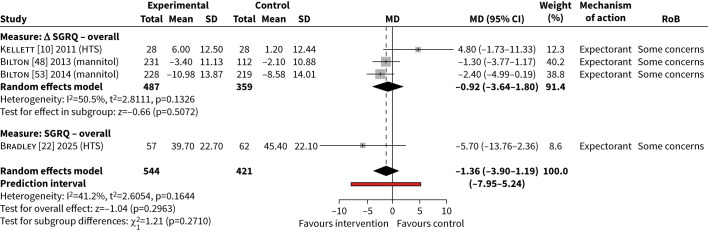
Meta-analysis of St. George's Respiratory Questionnaire (SGRQ) scores between mucoactive treatment and control groups in bronchiectasis. SGRQ overall scores range from 0 to 100, with higher scores indicating greater limitations. Red line indicates prediction interval, reflecting expected range of effects in a new study, accounting for between-study differences. MD: mean difference; HTS: hypertonic saline; RoB: overall risk of bias judgement;

For quality-of-life outcomes, meta-analysis of four randomised trials (937 patients) showed that mucoactive treatment was not associated with differences in total St. George's Respiratory Questionnaire (SGRQ) score (figure 4). The MD was –1.36 (95% CI –3.90–1.19; p=0.30; prediction interval –7.95–5.24), with moderate heterogeneity (I^2^=41.2%, p=0.16). A sensitivity analysis including carbocisteine data from Bradley *et al.* [22] (MD −5.00, 95% CI −12.56–2.56) instead of the trial's HTS data showed the overall MD was −1.35 (95% CI −3.81–1.11; p=0.28; prediction interval –7.62–4.92), also with moderate heterogeneity (I^2^=38.6%, p=0.18).

**FIGURE 5:**
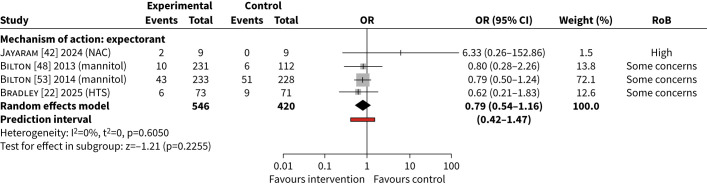
Meta-analysis of adverse events in patients with bronchiectasis following mucoactive treatment *versus* control. Proportions of patients with adverse events from four studies were analysed. Red line indicates prediction interval, reflecting expected range of effects in a new study, accounting for between-study differences. HTS: hypertonic saline; NAC: N-acetylcysteine;RoB: overall risk of bias judgement.

For safety outcomes, meta-analysis of four randomised trials (966 patients) showed that mucoactive treatment was not associated with differences in the odds of AEs (figure 5). The overall OR was 0.79 (95% CI 0.54–1.16; p=0.23; prediction interval 0.42–1.47), with no heterogeneity (I^2^=0%, p=0.61). A sensitivity analysis including carbocisteine data from Bradley *et al.* [22] (OR 1.13, 95% CI 0.43–2.97) instead of the trial's HTS data gave an overall OR of 0.86 (95% CI 0.59–1.25; p=0.43; prediction interval 0.46–1.59), with no heterogeneity (I^2^=0%, p=0.57).

GRADE assessment indicated that all outcomes were of “very low” certainty, except for FVC (L), sputum weight, hospital admissions, overall SGRQ score and total Leicester Cough Questionnaire (LCQ) score, which were “low” certainty (tables S7–S10).

Additional data on exacerbation duration, further spirometric parameters, sputum characteristics (sputum volume and weight), *Pseudomonas aeruginosa* status, hospital admissions and quality of life measures (including LCQ scores) are available in the supplemental material.

## Discussion

This review substantially updates earlier reviews of mucoactive agents in bronchiectasis [19, 21], incorporating 24 randomised and nonrandomised studies with 7051 participants. The evaluation covers eight mucoactive agents, including HTS and oral mucoactives. Meta-analyses reveal no statistically significant effects on exacerbations, sputum characteristics, quality of life or symptoms. While mucoregulator treatment marginally improved FEV_1_ % pred, this benefit was unlikely to be clinically meaningful and was not observed across other spirometry parameters, including FEV_1_ (L) [58]. Despite this isolated finding, when pooled, the evidence, predominantly of low quality, suggests that mucoactive agents do not demonstrate consistent or robust effects across key outcomes in routine bronchiectasis management. However, the effects of specific individual agents could not be fully distinguished due to the limited amount of trial evidence.

In a meta-analysis of seven randomised trials, mucoactive therapy was not associated with a reduction in annualised exacerbation incidence; however, the overall analysis demonstrated substantial statistical heterogeneity. Notably, two HTS studies reported conflicting results: one showing increased exacerbations [17] and another showing decreased exacerbations [10], inconsistencies which were also noted by Xie *et al.* [18] when studies were subgrouped by mechanism of action, heterogeneity was markedly lower among expectorants; however, there remained no significant treatment effect.

Interestingly, nonrandomised studies suggested larger reductions in annual exacerbation rates with mucoactive therapy than RCTs, highlighting important methodological differences. While observational studies better reflect real-world prescribing patterns, they are vulnerable to confounding by indication, as treatment allocation may be influenced by baseline symptom burden or exacerbation frequency and may therefore overestimate treatment effects. Clinicians may preferentially prescribe mucoactive agents to patients with greater sputum production or higher exacerbation risk, who have greater capacity for improvement. At the same time, such targeted prescribing may identify patients with higher disease activity who are more likely to benefit from therapy [59]. RCTs remain essential, as randomisation ensures treatment allocation is independent of baseline patient factors [16].

The rationale for mucoactive therapy has shifted as evidence has accumulated. Earlier guidelines recommended these agents primarily for exacerbation reduction [13, 14], while the 2025 ERS guidelines now prioritise improvements in quality of life and symptoms [15]. However, our meta-analysis demonstrates neither benefit; no association with a significant reduction in exacerbations or improvement in quality of life. Even when analyses were restricted *post hoc* to exacerbation enriched populations (≥2 exacerbations in the preceeding year) or RCTs enrolling patients with chronic sputum production, there was no significant reduction in exacerbation incidence. This progressive narrowing of evidence-supported indications raises questions about specific patient subgroups who may genuinely benefit.

This review incorporates a broad range of mucoactive agents with differing mechanisms of action. Whilst providing a large data pool, this inclusiveness introduces heterogeneity in drugs, doses and study settings, limiting interpretation. The small number of studies for each mucoactive prevented meaningful subgroup analyses by agent and inconsistent reporting of key outcomes and baseline characteristics (*e.g.* exacerbation history, mucus expectoration difficulty) (table S3) further limits conclusions about which patients may benefit most.

Differences in disease aetiology and severity, comorbidities, and clinical practice among patient populations included in this review may contribute to the observed lack of efficacy of mucoactive treatments [7]. The “treatable traits” concept introduced by Boaventura *et al.* [60] could improve future trial outcomes by identifying optimal populations for mucoactive treatment based on underlying biology. Patients with pulmonary traits such as mucus hypersecretion may benefit more with mucoactives than those with etiological traits such as primary immunodeficiencies. Supporting this view, Gao *et al.* [61] conducted a *post hoc* analysis of a “negative” mannitol randomised trial (included study: Bilton *et al.* [53]), uncovering that targeting highly symptomatic patients may reveal treatment efficacy; 32.7% of those in the inhaled mannitol group with a high symptom burden experienced no exacerbations compared to 14.6% in the control group.

However, inconsistent baseline characterisation across studies limited our ability to evaluate precision approaches in our review. Only five of 25 studies reported patient aetiology data and only five reported bronchiectasis severity using validated multidimensional scores (including the Bronchiectasis Severity Index and FACED (FEV_1_, age, chronic colonisation, extension, dyspnoea)) (table S4). This represents a critical gap preventing evidence-based patient stratification. While the 2025 ERS guidelines suggest targeting highly symptomatic patients based on the *post hoc* analysis by Gao *et al.* [61], only six studies across our comprehensive review reported baseline symptom scores using validated instruments (including the Quality of Life Questionnaire–Bronchiectasis, SGRQ and LCQ) [15, 61]. This underscores the urgent need for standardised “treatable trait” reporting in future bronchiectasis trials to enable identification of patients (if any) most likely to benefit from mucoactive therapy, including symptom burden, disease severity and aetiology. Variation in trial inclusion criteria may also contribute to heterogeneity in treatment effects; for example, studies differed in exacerbation thresholds and sputum-related eligibility criteria (table S4). Additionally, variation in co-intervention reporting, particularly the use of ACTs, may also have influenced observed treatment effects across trials. In most studies ACTs were either permitted as part of usual care or not explicitly described and details regarding type, frequency or adherence were rarely reported (table S3), precluding meaningful sensitivity analyses.

While lack of success in trials has often been attributed to patient population heterogeneity, Long *et al.* [62] argue that the expected benefits of mucoactive agents may have been overestimated in bronchiectasis. Many mucoactive therapies were developed for CF, where defective CF transmembrane conductance regulator (CFTR)-mediated ion transport causes airway-surface liquid depletion and uniformly hyper-concentrated mucus with impaired mucociliary clearance [63, 64]. Mucociliary clearance is also impaired in bronchiectasis and secretions are hyper-concentrated compared to healthy airways. However, these abnormalities are typically secondary to infection-driven neutrophilic inflammation rather than a primary ion transport defect and vary between patients [64].

This biological heterogeneity may contribute to inconsistent trial findings and supports identifying patients with greater sputum burden or demonstrable mucus clearance impairment within a “treatable traits” framework [64]. However, a 2023 Cochrane review reported low to very low certainty evidence for HTS even in CF, questioning the robustness of these therapeutic approaches [65]. Consistent with this, the recent SIMPLIFY trial showed that CF patients receiving effective CFTR modulators could discontinue HTS and rhDNAse without short-term harm, with longer-term withdrawal of mucoactive agents currently under investigation [66, 67].

The overall risk of bias was high for most randomised trials (12/21). While including observational cohort studies increased representativeness, it introduced greater potential for bias, with three of four studies rated as “serious risk” or lacking sufficient information for assessment. Consequently, the overall certainty of evidence in this review was graded as “low” or “very low” for all outcomes. Bronchiectasis trials face inherent challenges; for example, exacerbations are uncommon, requiring high baseline event rates to detect effects [63], lung function declines slowly, necessitating long-duration studies, and sputum production is subjectively perceived by patients [63]. Selection of an appropriate placebo represents an additional challenge, as isotonic saline, commonly used as a control, may itself improve airway hydration and mucus clearance, potentially reducing apparent treatment effect size [17]. A sensitivity analysis excluding studies using potentially active comparators (*e.g.* isotonic saline or low-dose mannitol) showed no reduction in exacerbation incidence.

These methodological limitations, including difficulty with blinding due to taste differences between active and placebo solutions [65], and the need for larger, longer duration studies, may partly explain the weak evidence base observed. In addition, variation in follow-up duration across studies may contribute to heterogeneity in pooled lung function and quality of life outcomes.

In summary, pooled evidence from current trials did not demonstrate consistent benefits of mucoactive agents across key outcomes. The recently published 2025 ERS guidelines maintain a conditional recommendation for mucoactive treatments with very low certainty evidence, even weaker than the 2017 “low quality” assessment [15]. Our systematic review, including 25 studies with 7075 participants, provides the most comprehensive evidence synthesis to date and validates this cautious approach. Importantly, our review incorporates real-world observational data and robust 52-week findings for both HTS and carbocisteine from the recent CLEAR trial [22].

With no demonstrated associations with reduced exacerbations or quality of life benefits, the 28% of bronchiectasis patients currently using mucoactive agents could potentially be receiving burdensome and unnecessary therapy [7]. Given the very low certainty of evidence, further trials are needed to clarify which specific patients, if any, may benefit from particular mucoactive agents.

**Points for clinical practice**

**Evidence before this study**

A 2014 Cochrane systematic review of mucolytics in bronchiectasis included four trials with mixed findings. Bromhexine with antibiotics during exacerbations improved sputum clearance, while erdosteine produced small short-term spirometry gains. Recombinant human DNase (rhDNase) showed harm without benefit. Evidence was insufficient to support routine mucolytic use, and hypertonic saline was not evaluated.

A 2017 systematic review of inhaled mucoactive agents in chronic non-CF lung diseases confirmed harm with rhDNase in bronchiectasis but found hypertonic and normal saline improved lung function, quality of life and sputum burden. However, most bronchiectasis trials were small, short duration and heterogeneous, limiting conclusions about long-term efficacy.

The 2025 ERS guidelines evaluated nine randomised trials of mucoactive treatments in bronchiectasis. The meta-analysis found statistically significant improvements in quality of life (SGRQ: MD −2 points, 95% CI −3.6– −0.4; three studies) and symptoms (one study), but exacerbation outcomes varied across studies. Based on this evidence, the ERS issued a conditional recommendation to offer mucoactive treatments to patients where airway clearance has failed to control symptoms.

**Added value of this study**

Several large studies have been published more recently, making an updated evidence synthesis timely and necessary.

We searched Medline, Embase, Cochrane CENTRAL, EU, ClinicalTrials.gov and WHO trial registries, and relevant reference lists to 1 October 2025. Eligible studies included adults with bronchiectasis, excluding paediatric and CF populations. This review substantially updates the evidence base, incorporating 24 randomised and nonrandomised studies with 7051 participants across eight mucoactive agents.

**Implications of all the available evidence**

Patients have emphasised the need for robust evaluation of mucoactive agents. Approximately three-quarters of bronchiectasis patients may be missing an affordable therapy targeting key symptoms and reducing exacerbations if these agents are effective. Conversely, the one-quarter currently using them may be receiving unnecessary treatment if ineffective.

Our meta-analyses did not demonstrate statistically significant effects of mucoactive agents (ambroxol, bromhexine, carbocisteine, erdosteine, hypertonic saline, mannitol, N-acetylcysteine, rhDNase) on exacerbations, quality of life or symptoms. Although treatment was associated with improved FEV_1_ % pred, this was unlikely to be clinically meaningful and was not observed across other spirometry parameters.

Taken together, the available evidence, although predominantly of low quality, does not demonstrate consistent or robust effectiveness across key outcomes in routine bronchiectasis management. These findings validate the downgrading of evidence certainty in the 2025 ERS guidelines from “low” (2017) to “very low” (2025) and the maintenance of only conditional recommendations and provide the most comprehensive evidence base to date to inform clinical practice.

## Supplementary material

**Please note:** supplementary material is not edited by the Editorial Office, and is uploaded as it has been supplied by the author. Supplementary material ERR-0014-2026.SUPPLEMENT
