# Supplementary material for: Mucoactive agents in bronchiectasis: a systematic review and meta-analysis
Source: Eur Respir Rev. 2026 Jun 24;35(180):260014. doi: 10.1183/16000617.0014-2026 (PMC13291835; doi:10.1183/16000617.0014-2026)
Supplement: Supplementary file 1 [file ERR-0014-2026.SUPPLEMENT.pdf]

Supplemental appendix

Contents

Mucoactive agents 3
Search strategy 4
Characteristics of included studies 6
Participant inclusion criteria 14
Excluded studies 18
Ongoing studies 26
Risk of Bias 28
Additional Data 29
Exacerbation Duration 29
Lung Function 30
Forced Expiratory Volume in 1 second (FEV_1) 30
Forced Vital Capacity (FVC) 31
FVC Percentage Predicted 32
FEV_1/FVC Ratio 32
Sputum characteristics & microbiology 32
Sputum Volume 32
Sputum Weight 33
Pseudomonas aeruginosa status 33
Safety 34
Hospital Admissions 34
Quality of Life 34
Total LCQ Scores 34
Exercise Capacity 34
GRADE findings 36
Evidence profile 36
Summary of findings: exacerbations and lung function 40
Summary of findings: symptoms and sputum characteristics 43
Summary of findings: safety 45
Funnel Plots for Detecting Publication Bias (Meta-Analyses with >10 Studies) 46

Mucoactive agents

Table S1 - Mucoactive Medications: *Agents specifically designed to alter the viscoelastic properties of mucus and promote secretion clearance are categorised according to their mechanism of action. Some mucoactive agents may have multiple mechanisms of action. Isotonic saline, used as a placebo in several studies, is not considered an active mucoactive agent. Clinical uses in airway conditions are included alongside World Health Organisation Anatomical Therapeutic Chemical (ATC) classification.*

Category		Function	Subcategory	Drug name	Clinical use (ATC class)	Condition	Ongoing trials in bronchiectasis
**Mucolytics**	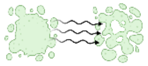	Break down mucus protein bonds or DNA to reduce viscosity and facilitate clearance	Classic mucolytics (thiol derivatives)	N-Aceylcysteine (NAC)	Yes (R05CB01)	Cough and common cold	ChiCTR2000031817, ChiCTR-TRC-13003792
				N-Acystelyn (NAL)	No		
				Fudosteine	Yes (Japan)	Bronchitis	
			Peptide mucolytics	rhDNase	Yes (R05CB13)	Cystic fibrosis	
				Gelsolin	No		
				Thymosin β4	No		
			Non-destructive mucolytics	Dextran	No		
				Heparin	No		
**Expectorants**	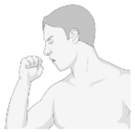	Increase mucus water content to enhance clearance		Hypertonic saline	Yes (B05CB01)	Cystic fibrosis	NCT04140214, NCT02392663, NCT06443658, NCT06242795
				Mannitol	Yes (R05CB16)	Cystic fibrosis	
				Guaifenesin	Yes (R05CA03)	Cough and common cold	
				Iodide-containing compounds	Yes (R05CA02)	Cough and common cold	
**Mucoregulators**	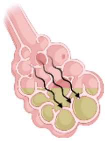	Regulate mucus production and secretion		Carbocysteine	Yes (R05CB03)	Cough and common cold	
				Erdosteine	Yes (R05CB15)	Bronchitis	ACTRN12621000315819
**Mucokinetics**	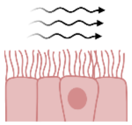	Improve mucus transport by enhancing ciliary activity and mucus flow	Surfactants	Beractant	Yes (R07AA)	Respiratory distress syndrome	
				Calfactant	No		
				Bromhexine	Yes (R05CB02)	Bronchitis	
				Ambroxol	Yes (R05CB06)	Bronchitis	ChiCTR2200066348

Search strategy

Table S2 - Example search strategy as used for MEDLINE. *Search strategy ran on 14th April 2025.*

#	Search Term
1	Bronchiectasis
2	"Non cystic fibrosis bronchiectasis"
3	"Non-CF bronchiectasis"
4	1 OR 2 OR 3
5	Expectorants
6	Acetylcysteine
7	Ambroxol
8	Bromhexine
9	Carbocysteine
10	Guaifenesin
11	"potassium citrate"
12	Mucolytic
13	Mucoactive
14	Mucokinetic
15	"Mucociliary clearance"
16	S-carboxymethylcysteine
17	Sobrerol
18	"Iodinated glycerol"
19	"Human Dnase"
20	DNase
21	RhDNase
22	ExoDeoxyribonucleases
23	"Dornase alfa"
24	Pulmozyme
25	"Sodium Chloride"
26	NaCl
27	"Hypertonic saline solution"
28	"Hypertonic saline"
29	HTS
30	Saline
31	"Sodium bicarbonate"
32	Carbocisteine
33	N-acetylcysteine
34	NAC
35	Methocarbamol
36	"Ammonium chloride"
37	Sodium citrate
38	Guaiphenesin
39	Guaifenesin

40	"Glyceryl guaiacolate"
41	Erdosteine
42	Mecysteine
43	Mannitol
44	"Mannitol phosphates"
45	Mitobronitol
46	Mesna
47	"2-Mercapto ethane sodium sulfonate"
48	"Potassium dichromate"
49	Guaiacolsulfonate
50	Guaiacolsulphonate
51	Sulfoguaiacolum
52	Tyloxapol
53	Stepronin
54	Heparin
55	Beclomethasone-formoterol
56	Beclomethasone
57	Formoterol
58	Hyaluronic acid
59	5 OR 6 OR 7 OR 8 OR 9 OR 10 OR 11 OR 12 OR 13 OR 14 OR 15 OR 16 OR 17 OR 18 OR 19 OR 20 OR 21 OR 22 OR 23 OR 24 OR 25 OR 26 OR 27 OR 28 OR 29 OR 30 OR 31 OR 32 OR 33 OR 34 OR 35 OR 36 OR 37 OR 38 OR 39 OR 40 OR 41 OR 42 OR 43 OR 44 OR 45 OR 46 OR 47 OR 48 OR 49 OR 50 OR 51 OR 52 OR 53 OR 54 OR 55 OR 56 OR 57 OR 58
60	"Randomized controlled trial"
61	"Randomised controlled trial"
62	"Randomised trial"
63	"Randomized trial"
64	"RCT"
65	"Controlled clinical trial"
66	"Controlled study"
67	"Clinical trial"
68	"Clinical study"
69	"Comparative study"
70	"Evaluation study "
71	"Prospective study"
72	"Longitudinal study"
73	"Observational study"
74	"Cohort Study"
75	"Non-randomised study"
76	"Cross-sectional study"
77	"Case-control study"
78	"Prospective study"
79	60 OR 61 OR 62 OR 63 OR 64 OR 65 OR 66 OR 67 OR 68 OR 69 OR 70 OR 71 OR 72 OR 73 OR 74 OR 75 OR 76 OR 77 OR 78
80	4 AND 59 AND 79

Characteristics of included studies

Table S3 - Characteristics of included studies. **Only abstract available. †Pooled Mean±SD. Mucoactive agents category colour key: orange = expectorants; yellow = mucoregulators; green = mucokinetics; blue = mucolytics. Concomitant therapies column includes airway clearance technique (ACT) information in square brackets, classified as: protocolised (ACT specified in the study protocol), permitted (allowed as usual care but not standardised), restricted (background ACT not permitted or replaced by the study intervention), or not reported.*

#	Author(s) and Year	Mucoactive agent (intervention and dose)	Comparator	Outcomes measured relevant for this review	Region	Study Design	Duration to Last Follow-up (Weeks)	Diagnostic Criteria	Sample Size	Age (Years, Mean±SD)	% Male/Female	Number of Withdrawals	Reasons for Withdrawal	Challenge Test Conducted	Concomitant Therapies [ACT use]
1	Basavaraj *et al.*, 2024 [1]	Nebulised hypertonic saline	± positive expiratory pressure, no airway clearance management	Pulmonary exacerbations, admissions to hospital, lung function, cough, sputum microbiology.	USA	Non-randomised	N/A	High-resolution CT scan, cough and/or daily mucopurulent phlegm	2195	67.5±12.4	21/79	3152 excluded from original 5346	No data available for analysis, documented diagnosis of CF, no bronchiectasis diagnosis/no CT scan, receiving other forms of chest physiotherapy, missing/conflicting airway clearance data	No	Bronchial hygiene measures other than HS or PEP excluded [Permitted (usual care)]
2	Bilton *et al.*, 2013 [2]	Inhaled mannitol (320mg)	Inhaled mannitol (10mg)	Pulmonary exacerbations, admissions to hospital, lung function, quality of life, mucus characteristics, sputum microbiology, cough, shortness of breath, exercise tolerance, adverse events, patients' perception of health.	Australia, New Zealand, UK	Randomised	12	High-resolution CT scan	362	61.9±9.4†	Not reported	57	AEs, lost to follow up, patient decision, eligibility criteria not met, physician decision, positive airway challenge, sponsor decision, other	Yes	Antibiotics [Permitted (usual care)]
3	Bilton *et al.*, 2014 [3]	Inhaled mannitol (400mg)	Inhaled mannitol (50mg)	Pulmonary exacerbations, lung function, quality of life, mucus characteristics.	USA, Europe, Australia, New Zealand, South America	Randomised	52	High-resolution CT scan	485	59.5±13.5†	37.4/62.6	81	Withdrew consent, adverse events, lost to follow up, protocol deviations, died, physician decision	Yes	Nebulised HTS prohibited, all other therapies continued [Permitted (usual care)]

#	Author(s) and Year	Mucoactive agent (intervention and dose)	Comparator	Outcomes measured relevant for this review	Region	Study Design	Duration to Last Follow-up (Weeks)	Diagnostic Criteria	Sample Size	Age (Years, Mean±SD)	% Male/Female	Number of Withdrawals	Reasons for Withdrawal	Challenge Test Conducted	Concomitant Therapies [ACT use]
4	Bradley *et al.*, 2011 [4]*	Nebulised 6% hypertonic saline	0.9% isotonic saline	Quality of life	Northern Ireland	Randomised, crossover	4	CT scan, clinical features	19	Not reported	Not reported	Not reported	N/A	Not reported	Not reported [Not reported]
5	Bradley *et al.*, 2025 [5]	Nebulised 6% hypertonic saline<br>carbocisteine (750mg)	Standard care	Pulmonary exacerbations, lung function, quality of life, adverse events	UK	Randomised, **2x2 factorial**	52	High-resolution CT scan	288	65.7± 13.1[†]	40.6/59.4	21	Lost to follow up, protocol deviations, withdrew consent, AEs, death	Yes	Not reported [Permitted (usual care)]
6	Crisafulli *et al.*, 2007 [6]	Oral erdosteine (225mg)	Physiotherapy alone	Lung function, quality of life, mucus characteristics, cough, shortness of breath, exercise tolerance.	Italy	Randomised	2	CT scan, clinical features	30	71±11	70/30	Not reported	N/A	No	Patients excluded if used antibiotics, mucolytics, systemic steroids, or antitussive drugs, or if reported change in chronic medications in 4 weeks prior to study [Protocolised]
7	Daviskas *et al.*, 1999 [7]	Inhlaed mannitol (320mg)	Placebo	Cough	Australia	Randomised, crossover	<1	CT scan, bronchography	11	52±2	27.3/ 72.7	Not reported	N/A	Yes	inhaled b2-adrenergic agonists, glucocorticosteroids , antibiotics, nebulised gentamicin [Permitted (usual care)]
8	Daviskas *et al.*, 2008 [8]	Inhlaed mannitol (320mg, 160mg)	Placebo	Lung function	Australia	Randomised, crossover	<1	High-resolution CT scan	14	63±3	21.4/ 78.6	Not reported	N/A	Yes	Salbutamol, Ciclesonide, Flixotide, Doxycycline, Terbutaline, Salmetrol, Symbicort, Nuelin [Not reported]
9	Daviskas *et al.*, 2010 [9]	Inhlaed mannitol (635mg, 480mg, 320mg, 160mg)	Control	Mucus characteristics	Australia	Randomised, crossover	<1	High-resolution CT scan	14	63±3	Not reported	Not reported	N/A	Yes	None [Not reported]

#	Author(s) and Year	Mucoactive agent (intervention and dose)	Comparator	Outcomes measured relevant for this review	Region	Study Design	Duration to Last Follow-up (Weeks)	Diagnostic Criteria	Sample Size	Age (Years, Mean±SD)	% Male/Female	Number of Withdrawals	Reasons for Withdrawal	Challenge Test Conducted	Concomitant Therapies [ACT use]
10	Herrero-Cortina *et al.*, 2018 [10]	Nebulised 7% hypertonic saline	± 0.1% hyaluronic acid, 0.9% isotonic saline	Lung function, quality of life, mucus characteristics, cough, adverse events.	Spain	Randomised, crossover	3	High-resolution CT scan	28	64.0±17.5	35.7 / 64.3	5	Pulmonary exacerbation, new medical/personal condition	Yes	Bronchodilator (e.g. 200ug albuterol) each visit. Pharmacological treatments and medications remained unchanged. [Protocolised]
11	Ignatova *et al.*, 2019 [11]*	Nebulised hypertonic saline plus hyaluronic acid	SoC	Shortness of breath	Russian Federation	Non-randomised	4	Not Reported	99	59.3±24.2[†]	26.3/74.2	Not reported	N/A	Not reported	Not reported [Not reported]
12	Jayaram *et al.*, 2024 [12]	Inhaled N-acetylecysteine (1.2g)	Placebo	Lung function, quality of life, adverse events.	Australia	Randomised	6	CT scan	24	67.3±10.5	52.9/47.1	7	Disruption due to COVID pandemic	No	Combined inhaler therapy (ICS/LABA) [Not reported]
13	Kellett *et al.*, 2005 [13]	Nebulised 7% hypertonic saline	0.9% isotonic saline	Lung function, mucus characteristics, cough.	UK	Randomised, crossover	1	High-resolution CT scan	23	57.6±3.7	29.2/ 70.8	1	Lost to follow up	Yes	Nebulised terbutaline was used as a premedication. Patients continued medications: inhaled/nebulised bronchodilators, long acting bronchodilators, leukotriene antagonists, oral or inhaled steroids. [Protocolised]
14	Kellett *et al.*, 2011 [14]	Nebulised 7% hypertonic saline	0.9% isotonic saline	Pulmonary exacerbations, lung function, quality of life.	UK	Randomised, crossover	12	High-resolution CT scan	32	56.6±14.6	50/50	4	Hyper-responsive to HTS, new medical diagnosis, failure to comply with study visits	Yes	Chronic medications, medications for exacerbations as perceived appropriate (diary recorded) [Not reported]
15	Minov *et al.*, 2019 [15]	Carbocisteine (750mg)	Control	Pulmonary exacerbations, sputum microbiology.	Macedonia	Non-randomised	12	High-resolution CT scan	64	54.1±8.1	57.8/42.2	Not reported	N/A	No	None [Not reported]

#	Author(s) and Year	Mucoactive agent (intervention and dose)	Comparator	Outcomes measured relevant for this review	Region	Study Design	Duration to Last Follow-up (Weeks)	Diagnostic Criteria	Sample Size	Age (Years, Mean±SD)	% Male/Female	Number of Withdrawals	Reasons for Withdrawal	Challenge Test Conducted	Concomitant Therapies [ACT use]
16	Nicolson *et al.*, 2012 [16]	Nebulised 6% hypertonic saline	0.9% isotonic saline	Pulmonary exacerbations, admissions to hospital, lung function, quality of life, cough, sputum microbiology.	Australia	Randomised	52	High-resolution CT scan	40	57±15[†]	37.5/62.5	10	Did not meet criteria, depression, declined further treatment	Yes	200ug salbutamol. No restrictions on prescribed medications. [Restricted]
17	O'Donnell *et al.*, 1998 [17]	Nebulised rhDNase	Placebo	Pulmonary exacerbations, admissions to hospital, lung function.	North America, GB and Ireland	Randomised	24	CT scan or radiograph	349	Not reported	37.8/62.2	6	Death, haemoptysis, sinus symptoms	Not reported	Patients continued to receive usual care [Permitted (usual care)]
18	Olivieri *et al.*, 1991 [18]	Oral bromhexine (30mg)	Placebo	Lung function	Italy	Randomised	2	CT scan or bronchography	88	51.8±2.5[†]	64.8/35.2	Not reported	N/A	Not reported	Antibiotic treatment with or without bromhexine. During first week all patients were given ceftazidine.[Not reported]
19	Osculllo *et al.*, 2025 [19]	Oral N-acetylecysteine (600mg, 1200mg)	Control	Pulmonary exacerbations, admissions to hospital, mucus characteristics, sputum microbiology	Spain	Non-randomised	104	High-resolution CT scan	2630	64.5±16.0[†]	36.2/63.8	169	Diagnosis of CF	Not reported	Inhaled corticosteroids, long acting B-agonists [Permitted (usual care)]
20	Qi *et al.*, 2019 [20]	Oral N-acetylecysteine (600mg)	Control	Pulmonary exacerbations, lung function, quality of life, mucus characteristics, shortness of breath.	China	Randomised	52	High-resolution CT scan	161	54.9±12.2	39.7/60.3	22	Lost to follow up, deaths, unable to continue, deterioration, AEs	No	Inhaled corticosteroids, long acting B-agonists, inhaled short acting B-agonists, inhaled anticholinergics, inhaled corticosteroids, prednisone, theophylline [Not reported]
21	Rutland *et al.*, 1982 [21]	Oral carbocisteine (2.25g)	Placebo	Lung function	Australia	Randomised	12	Bronchography	40	Not reported	Not reported	Not reported	N/A	No	Any mucolytics and antitussives were discontinued and other therapy was rationalised.[Not reported]

#	Author(s) and Year	Mucoactive agent (intervention and dose)	Comparator	Outcomes measured relevant for this review	Region	Study Design	Duration to Last Follow-up (Weeks)	Diagnostic Criteria	Sample Size	Age (Years, Mean±SD)	% Male/Female	Number of Withdrawals	Reasons for Withdrawal	Challenge Test Conducted	Concomitant Therapies [ACT use]
22	Shehzad *et al.*, 2019 [22]	Inhaled N-acetylecysteine	Nebulised 3% hypertonic saline	Mucus characteristics	Pakistan	Randomised	<1	Acute exacerbation diagnosis	136	42.5±4.1	61.8/ 38.2	Not reported	N/A	Not reported	SoC treatment for bronchiectasis provided [Permitted (usual care)]
23	Wills *et al.*, 1996 [23]	Nebulised rhDNase (2.5mg)	Placebo	Lung function, quality of life, admissions to hospital, mucus characteristics, patients' perception of health.	England	Randomised	4	CT scan or bronchography	61	Not reported	45.9 /54.1	3	Consent withdrawn, dyspnea, hospitalisation	No	Other inhaled medications and chest physiotherapy, continued all other routine treatments for chest and other illnesses [Permitted (usual care)]
24	Zhong *et al.*, 2020 [24]	Oral ambroxol hydrochloride (30mg)	0.9% isotonic saline	Pulmonary exacerbations, lung function, quality of life, mucus characteristics, exercise tolerance.	China	Randomised, crossover	12	CT scan, clinical features	27	Not reported	48/52	8	Lost to follow up	No	Existing NCFB therapy [Permitted (usual care)]

1. Basavaraj A, Brunton AE, Choate R, et al. Nebulized hypertonic saline and positive expiratory pressure device use in patients with bronchiectasis: Analysis from the United States Bronchiectasis and NTM research registry. Respir Med Res 2024;86:101107.

2. Bilton D, Daviskas E, Anderson SD, et al. Phase 3 Randomized Study of the Efficacy and Safety of Inhaled Dry Powder Mannitol for the Symptomatic Treatment of Non-Cystic Fibrosis Bronchiectasis. Chest 2013;144(1):215–25.

3. Bilton D, Tino G, Barker AF, et al. Inhaled mannitol for non-cystic fibrosis bronchiectasis: a randomised, controlled trial. Thorax 2014;69(12):1073–9.

4. Bradley JM, Treacy K, O'Neill B, et al. S106 A randomised double blind 13 week crossover trial of hypertonic saline (HTS) (6%) vs isotonic saline (ITS) (0.9%) in patients with bronchiectasis. Thorax 2011;66(Suppl 4):A49–A49.

5. Bradley JM, O'Neill B, McAuley DF, et al. Hypertonic Saline or Carbocisteine in Bronchiectasis. N Engl J Med 2025;

6. Crisafulli E, Coletti O, Costi S, et al. Effectiveness of erdosteine in elderly patients with bronchiectasis and hypersecretion: A 15-day, prospective, parallel, open-label, pilot study. Clin Ther 2007;29(9):2001–9.

7. Daviskas E, Anderson SD, Eberl S, Chan H-K, Bautovich G. Inhalation of Dry Powder Mannitol Improves Clearance of Mucus in Patients with Bronchiectasis. Am J Respir Crit Care Med 1999;159(6):1843–8.

8. Daviskas E, Anderson SD, Eberl S, Young IH. Effect of increasing doses of mannitol on mucus clearance in patients with bronchiectasis. Eur Respir J 2008;31(4):765–72.

9. Daviskas E, Anderson SD, Young IH. Effect of mannitol and repetitive coughing on the sputum properties in bronchiectasis. Respir Med 2010;104(3):371–7.

10. Herrero-Cortina B, Alcaraz V, Vilaró J, Torres A, Polverino E. Impact of Hypertonic Saline Solutions on Sputum Expectoration and Their Safety Profile in Patients with Bronchiectasis: A Randomized Crossover Trial. J Aerosol Med Pulm Drug Deliv 2018;31(5):281–9.

11. Ignatova G, Antonov V. Prospective Monitoring of Patients with Bronchiectasis [Internet]. In: C105. BRONCHIECTASIS: FROM CLINICAL PHENOTYPES TO TREATMENT. American Thoracic Society; 2019 [cited 2024 Oct 24]. p. A5703–A5703.Available from: https://www.atsjournals.org/doi/10.1164/ajrccm-conference.2019.199.1_MeetingAbstracts.A5703

12. Jayaram L, King PT, Hunt J, et al. Evaluation of high dose N- Acetylcysteine on airway inflammation and quality of life outcomes in adults with bronchiectasis: A randomised placebo-controlled pilot study. Pulm Pharmacol Ther 2024;84:102283.

13. Kellett F, Redfern J, Niven RM. Evaluation of nebulised hypertonic saline (7%) as an adjunct to physiotherapy in patients with stable bronchiectasis. Respir Med 2005;99(1):27–31.

14. Kellett F, Robert NM. Nebulised 7% hypertonic saline improves lung function and quality of life in bronchiectasis. Respir Med 2011;105(12):1831–5.

15. Minov J, Stoleski S, Petrova T, Vasilevska K, Mijakoski D, Karadzinska-Bislimovska J. Effects of A Long-Term Use of Carbocysteine on Frequency and Duration of Exacerbations in Patients with Bronchiectasis. Open Access Maced J Med Sci 2019;7(23):4030–5.

16. Nicolson CHH, Stirling RG, Borg BM, Button BM, Wilson JW, Holland AE. The long term effect of inhaled hypertonic saline 6% in non-cystic fibrosis bronchiectasis. Respir Med 2012;106(5):661–7.

17. O’Donnell AE, Barker AF, Ilowite JS, Fick RB. Treatment of Idiopathic Bronchiectasis With Aerosolized Recombinant Human DNase I. Chest 1998;113(5):1329–34.

18. Olivieri D, Ciaccia A, Marangio E, Marsico S, Todisco T, Del Vita M. Role of bromhexine in exacerbations of bronchiectasis. Double-blind randomized multicenter study versus placebo. Respir Int Rev Thorac Dis 1991;58(3–4):117–21.

19. Oscullo G, Méndez R, Olveira C, et al. Effect of N-Acetylcysteine on Bronchiectasis in a Real-life Study. Data From the Spanish RIBRON Registry. Arch Bronconeumol 2025;61(4):196–202.

20. Qi Q, Ailiyaer Y, Liu R, et al. Effect of N-acetylcysteine on exacerbations of bronchiectasis (BENE): a randomized controlled trial. Respir Res 2019;20(1):73.

21. Rutland J, Marriott C, Cole PJ. A Study of the Activity of Mucodyne in Bronchiectasis. Mucoregulation Respir Tract Disord 1982;(5):10–6.

22. Shehzad MI, Mannan MAU, Masood Alam, Abdul Rauf, Muhammad Imran Sharif. Airway Clearance in Bronchiectasis: A Randomized Control Trial of N-Acetylcysteine with 3% hypertonic saline. J Islamabad Med Dent Coll 2019;8(3):146–50.

23. Wills PJ, Wodehouse T, Corkery K, Mallon K, Wilson R, Cole PJ. Short-term recombinant human DNase in bronchiectasis. Effect on clinical state and in vitro sputum transportability. Am J Respir Crit Care Med 1996;154(2 Pt 1):413–7.

24. Zhong L, Xiong Y, Zheng Z, et al. Effect of short-term inhalation of warm saline atomised gas on patients with non-cystic fibrosis bronchiectasis. ERJ Open Res 2020;6(1):00130–2019.

Participant inclusion criteria

Table S4 – Summarised participant inclusion criteria of included studies.

#	Author(s) and Year	Exacerbation History	Sputum Production	Stability Period	Lung Function	Smoker Exclusion Criteria
1	Basavaraj *et al.,* 2024 [1]	Not reported	Cough and/or daily or frequent mucopurulent phlegm	Not reported	Not reported	Not reported
2	Bilton *et al.,* 2013 [2]	Not reported	Chronic sputum (>10mL/d) for majority of 3 mo	Clinically stable (for ≥2 wk prior to study entry)	FEV_1 ≥50% predicted and ≥1.0L	≥20 pack-y or >1 cigarette/wk within the previous 3 mo
3	Bilton *et al.,* 2014 [3]	Clinician documented history of at least two pulmonary exacerbations, each requiring antibiotic therapy, in the last 12 months and a total of at least four in the last two years	Production of ≥10g and 15mL/day sputum for majority of 3 mo	Not reported	FEV1 ≥ 40% and ≤85% predicted and ≥1.0L	Smoked within the last three months and must not smoke during participation in study
4	Bradley *et al.,* 2011 [4]*	Not reported	Not reported	Not reported	Not reported	Not reported
5	Bradley *et al.,* 2025	One or more pulmonary exacerbations in the last year requiring antibiotics. This can include patient reported exacerbations	Expectorate sputum on a daily basis and/or patients that expectorate sputum on most days but experience difficulty in expectoration on other days	Stable from a respiratory point of view for 14 or more days	Not reported	Current smokers, female ex-smokers with greater than 20 pack years and male ex-smokers with greater than 25 pack years
6	Crisafulli *et al*[X] 2007	No evidence of ongoing exacerbation as confirmed by medical history report and physical examination	Daily sputum >30 mL	Clinically stable	Not reported	Current smokers were excluded
7	Crisafulli *et al.,* 2007 [6]	Not reported	Not reported	Not reported	Not reported	Not reported
8	Daviskas *et al.,* 1999 [7]	Not reported	Not reported	Stable bronchiectasis	Not reported	Smokers and former smokers excluded
9	Daviskas *et al.,* 2008 [8]	Not reported	Not reported	Stable bronchiectasis	Not reported	Lifelong non-smokers only included
10	Daviskas *et al.,* 2010 [9]	Not reported	Spontaneous sputum expectoration mean ≥ 10g/24h	Clinically stable for a minimum of 4 weeks	FEV1 >30% after bronchodilation, total lung capacity >45%	Smokers or former smokers (>10-pack years)
11	Herrero-Cortina *et al.,* 2018 [10]	Not reported	Not reported	Not reported	Not reported	Not reported

12	Ignatova *et al.*, 2019 [11]*	Not reported	History of chronic sputum expectoration	Clinically stable for a minimum of 4 weeks	FEV1 >30% after bronchodilation, total lung capacity >45%	Current smoking or a smoking history of >20 pack years
13	Jayaram *et al.*, 2024 [12]	Not reported	Thick, sticky sputum	Stable bronchiectasis	Not reported	Not reported
14	Kellett *et al.*, 2005 [13]	Not reported	Not reported	Not reported	Not reported	Not reported
15	Kellett *et al.*, 2011 [14]	Not reported	Difficulties in expectorating sputum	Not reported	Not reported	Not reported
16	Minov *et al.*, 2019 [15]	At least 2 exacerbations requiring antibiotics per year for the previous 2 years	Daily sputum	Clinically stable	Not reported	Not reported
17	Nicolson *et al.*, 2012 [16]	Not reported	Daily purulent sputum production >15mL for the majority of 3 mo	Not reported	FEV1 >30% and <80% predicted	Not reported
18	O'Donnell *et al.*, 1998 [17]	Admitted with active exacerbation	Purulent expectoration (>20mL)	Active exacerbation (acute inflammatory infective stage)	Not reported	Not reported
19	Olivieri *et al.*, 1991 [18]	Complete data on exacerbations for two years of follow-up/before starting treatment	Not reported	Clinically stable	Not reported	Not reported
20	Oscullo *et al.*, 2025 [19]	At least two exacerbations in the past year	Not reported	Clinically stable for at least 4 weeks	Not reported	Current smokers, cigarette smoking within 6 months
21	Qi *et al.*, 2019 [20]	Not reported	At least 5mL sputum daily for 6 mo	Clinically stable	Not reported	Not reported
22	Rutland *et al.*, 1982 [21]	Diagnosed cases of acute exacerbation	Not reported	Not reported	Not reported	None reported
23	Shehzad *et al.*, 2019 [22]	Not reported	Not reported	Not reported	Not reported	Not reported
24	Wills *et al.*, 1996 [23]	Not reported	>10mL purulent sputum daily	Clinically stable	Not reported	Smoking history or had quit smoking for <1 year

1. Basavaraj A, Brunton AE, Choate R, et al. Nebulized hypertonic saline and positive expiratory pressure device use in patients with bronchiectasis: Analysis from the United States Bronchiectasis and NTM research registry. Respir Med Res 2024;86:101107.

2. Bilton D, Daviskas E, Anderson SD, et al. Phase 3 Randomized Study of the Efficacy and Safety of Inhaled Dry Powder Mannitol for the Symptomatic Treatment of Non-Cystic Fibrosis Bronchiectasis. Chest 2013;144(1):215–25.

3. Bilton D, Tino G, Barker AF, et al. Inhaled mannitol for non-cystic fibrosis bronchiectasis: a randomised, controlled trial. Thorax 2014;69(12):1073–9.

4. Bradley JM, Treacy K, O’Neill B, et al. S106 A randomised double blind 13 week crossover trial of hypertonic saline (HTS) (6%) vs isotonic saline (ITS) (0.9%) in patients with bronchiectasis. Thorax 2011;66(Suppl 4):A49–A49.

5. Bradley JM, O’Neill B, McAuley DF, et al. Hypertonic Saline or Carbocisteine in Bronchiectasis. N Engl J Med 2025;

6. Crisafulli E, Coletti O, Costi S, et al. Effectiveness of erdosteine in elderly patients with bronchiectasis and hypersecretion: A 15-day, prospective, parallel, open-label, pilot study. Clin Ther 2007;29(9):2001–9.

7. Daviskas E, Anderson SD, Eberl S, Chan H-K, Bautovich G. Inhalation of Dry Powder Mannitol Improves Clearance of Mucus in Patients with Bronchiectasis. Am J Respir Crit Care Med 1999;159(6):1843–8.

8. Daviskas E, Anderson SD, Eberl S, Young IH. Effect of increasing doses of mannitol on mucus clearance in patients with bronchiectasis. Eur Respir J 2008;31(4):765–72.

9. Daviskas E, Anderson SD, Young IH. Effect of mannitol and repetitive coughing on the sputum properties in bronchiectasis. Respir Med 2010;104(3):371–7.

10. Herrero-Cortina B, Alcaraz V, Vilaró J, Torres A, Polverino E. Impact of Hypertonic Saline Solutions on Sputum Expectoration and Their Safety Profile in Patients with Bronchiectasis: A Randomized Crossover Trial. J Aerosol Med Pulm Drug Deliv 2018;31(5):281–9.

11. Ignatova G, Antonov V. Prospective Monitoring of Patients with Bronchiectasis [Internet]. In: C105. BRONCHIECTASIS: FROM CLINICAL PHENOTYPES TO TREATMENT. American Thoracic Society; 2019 [cited 2024 Oct 24]. p. A5703–A5703.Available from: https://www.atsjournals.org/doi/10.1164/ajrccm-conference.2019.199.1_MeetingAbstracts.A5703

12. Jayaram L, King PT, Hunt J, et al. Evaluation of high dose N- Acetylcysteine on airway inflammation and quality of life outcomes in adults with bronchiectasis: A randomised placebo-controlled pilot study. Pulm Pharmacol Ther 2024;84:102283.

13. Kellett F, Redfern J, Niven RM. Evaluation of nebulised hypertonic saline (7%) as an adjunct to physiotherapy in patients with stable bronchiectasis. Respir Med 2005;99(1):27–31.

14. Kellett F, Robert NM. Nebulised 7% hypertonic saline improves lung function and quality of life in bronchiectasis. Respir Med 2011;105(12):1831–5.

15. Minov J, Stoleski S, Petrova T, Vasilevska K, Mijakoski D, Karadzinska-Bislimovska J. Effects of A Long-Term Use of Carbocysteine on Frequency and Duration of Exacerbations in Patients with Bronchiectasis. Open Access Maced J Med Sci 2019;7(23):4030–5.

16. Nicolson CHH, Stirling RG, Borg BM, Button BM, Wilson JW, Holland AE. The long term effect of inhaled hypertonic saline 6% in non-cystic fibrosis bronchiectasis. Respir Med 2012;106(5):661–7.

17. O'Donnell AE, Barker AF, Ilowite JS, Fick RB. Treatment of Idiopathic Bronchiectasis With Aerosolized Recombinant Human DNase I. Chest 1998;113(5):1329–34.

18. Olivieri D, Ciaccia A, Marangio E, Marsico S, Todisco T, Del Vita M. Role of bromhexine in exacerbations of bronchiectasis. Double-blind randomized multicenter study versus placebo. Respir Int Rev Thorac Dis 1991;58(3–4):117–21.

19. Oscullo G, Méndez R, Olveira C, et al. Effect of N-Acetylcysteine on Bronchiectasis in a Real-life Study. Data From the Spanish RIBRON Registry. Arch Bronconeumol 2025;61(4):196–202.

20. Qi Q, Ailiyaer Y, Liu R, et al. Effect of N-acetylcysteine on exacerbations of bronchiectasis (BENE): a randomized controlled trial. Respir Res 2019;20(1):73.

21. Rutland J, Marriott C, Cole PJ. A Study of the Activity of Mucodyne in Bronchiectasis. Mucoregulation Respir Tract Disord 1982;(5):10–6.

22. Shehzad MI, Mannan MAU, Masood Alam, Abdul Rauf, Muhammad Imran Sharif. Airway Clearance in Bronchiectasis: A Randomized Control Trial of N-Acetylcysteine with 3% hypertonic saline. J Islamabad Med Dent Coll 2019;8(3):146–50.

23. Wills PJ, Wodehouse T, Corkery K, Mallon K, Wilson R, Cole PJ. Short-term recombinant human DNase in bronchiectasis. Effect on clinical state and in vitro sputum transportability. Am J Respir Crit Care Med 1996;154(2 Pt 1):413–7.

24. Zhong L, Xiong Y, Zheng Z, et al. Effect of short-term inhalation of warm saline atomised gas on patients with non-cystic fibrosis bronchiectasis. ERJ Open Res 2020;6(1):00130–2019.

Excluded studies

Table S5 - Table of excluded studies.

Title	Authors/ID	Year	Exclusion reason
Erdosteine in children and adults with bronchiectasis (BETTER trial): study protocol for a multicentre, double-blind, randomised controlled trial.	Chang et al.	2024	Wrong study design
Comparative effectiveness and safety of inhaled corticosteroid plus long-acting beta2-agonist fixed-dose combinations vs. long-acting muscarinic antagonist in bronchiectasis.	Su et al.	2024	Wrong intervention
The effect of beclomethasone-formoterol versus placebo on chronic cough in patients with non-CF bronchiectasis: the FORZA randomised controlled trial.	van der Veer et al.	2023	Wrong intervention
Intravenous N-acetylcysteine in respiratory disease with abnormal mucus secretion.	Tang et al.	2023	Wrong patient population
[Effectiveness of nebulized hypertonic saline in patients with bronchial hypersecretion].	Gonzalez-Montaos et al.	2023	Wrong comparator
The effect of N-acetylcysteine in patients with non-cystic fibrosis bronchiectasis (NINCFB): study protocol for a multicentre, double-blind, randomised, placebo-controlled trial.	Liao et al.	2022	Ongoing study
Efficacy of N-acetylcysteine on idiopathic or postinfective non-cystic fibrosis bronchiectasis: a systematic review and meta-analysis protocol.	Luo et al.	2022	Wrong study design
Heterogeneity of treatment response in bronchiectasis clinical trials.	Sibila et al.	2022	Wrong study design
Effects of a Mixture of Ivy Leaf Extract and Coptidis rhizome on Patients with Chronic Bronchitis and Bronchiectasis.	Hong et al.	2021	Wrong comparator
Therapeutic effect of nebulized hypertonic saline for muco-obstructive lung diseases: a systematic review and meta-analysis with trial sequential analysis.	Zhang et al.	2021	Wrong study design
Employment of an algorithm of care including chest physiotherapy results in reduced hospitalizations and stability of lung function in bronchiectasis.	Powner et al.	2019	Wrong intervention
Predicting factors for chronic colonization of Pseudomonas aeruginosa in bronchiectasis.	Pieters et al.	2019	Wrong study design
Addition of hyaluronic acid improves tolerance to 7% hypertonic saline solution in bronchiectasis patients.	Maiz et al.	2018	Wrong study design

Title	Authors/ID	Year	Exclusion reason
A randomised controlled trial on the effect of inhaled hypertonic saline on quality of life in primary ciliary dyskinesia.	Paff et al.	2017	Wrong patient population
Inhaled hyperosmolar agents for bronchiectasis.	Hart et al.	2014	Wrong study design
Efficacy of Halotherapy for Improvement of Pulmonary function Tests and Quality of Life of Non-Cystic Fibrosis Bronchiectatic Patients.	Rabbani et al.	2013	Wrong comparator
Sodium cromoglycate and eformoterol attenuate sensitivity and reactivity to inhaled mannitol in subjects with bronchiectasis.	Briffa et al.	2011	Wrong intervention
The 24-h effect of mannitol on the clearance of mucus in patients with bronchiectasis.	Daviskas et al.	2001	Wrong study design
Influence of a fluidifying agent (bromhexine) on the penetration of antibiotics into respiratory secretions.	Bergogne-Berezin et al.	1985	Wrong outcomes
Clinical effects of proteinase, sfericase (AI-794), on chronic bronchitis and similar diseases.	Itoh et al.	1984	Wrong intervention
The use and efficacy of mucolytic agents.	Benjamin et al.	1971	Wrong comparator
A new mucolytic, bromhexine ("bisolvon"). A double-blind study.	Bateman et al.	1971	Wrong patient population
NEBULISED MEDICATIONS IN SECONDARY CARE: RISING TO THE CHALLENGE	Bettany et al.	2022	Wrong study design
EVALUATION OF A NOVEL AIRWAY CLEARANCE SYSTEM IN PATIENTS WITH BRONCHIECTASIS	MINGORA et al.	2023	Wrong intervention
Evaluation of safety and efficacy of inhaled ambroxol in hospitalized adult patients with mucopurulent sputum and expectoration difficulty	Zheng et al.	2023	Wrong patient population
Advances in pharmacotherapy for bronchiectasis in adults	Zhang et al.	2023	Wrong study design
Clinical study of Eucalyptol, Limonene and Pinene Enteric Soft Capsules combined with piperacillin tazobactam in treatment of bronchiectasis	Zuo et al.	2021	Wrong intervention
Clinical Effect of Qingbu Weijing Decoction on Patients Suffering from Stable Bronchiectasis Complicated with Airway Mucus Hypersecretion	Yuan et al.	2023	Wrong intervention
Tolerance and safety of inhaled hypertonic saline solutions in patients with chronic bronquial infection	Velasco et al.	2022	Wrong patient population
A study to monitor adverse drug reactions in patients of chronic obstructive pulmonary disease, asthma	Maurya et al.	2022	Wrong study design

Title	Authors/ID	Year	Exclusion reason
and bronchiectasis			
Bronchiectasis and Gender: Results from the National Bronchiectasis and NTM Research Registry	Kang et al.	2022	No full text and no outcome data in abstract
Clinical characteristics of patients with post-tuberculosis bronchiectasis: Findings from the KMBARC registry	Choi et al.	2021	Wrong study design
Nebulized saline treatment in patients with concomitant asthma and bronchiectasis	Perez-Urria et al.	2021	Wrong patient population
RCT Abstract- The efficacy and safety of colistimethate sodium delivered via the I-neb in bronchiectasis: the PROMIS-I randomized controlled trial	Haworth et al.	2021	Wrong intervention
The efficacy of inhaled hypertonic saline for bronchiectasis: a meta-analysis of randomized controlled studies	Xie et al.	2020	Wrong study design
Bronchiectasis in Italy: data from the national registry IRIDE	Aliberti et al.	2020	Wrong study design
Identifying inconsistences in inpatient bronchiectasis management at a large university teaching hospital	Crowley et al.	2020	No full text and no outcome data in abstract
Heterogeneity of treatment response in bronchiectasis clinical trials	Sibila et al.	2020	Wrong study design
CLINICAL OUTCOMES AND HEALTHCARE RESOURCE UTILIZATION IN ADULT HIGH-RISK PATIENTS WITH NONCYSTIC FIBROSIS BRONCHIECTASIS USING HIGH FREQUENCY CHEST WALL OSCILLATION AND POSITIVE EXPIRATORY PRESSURE THERAPY	Basavaraj et al.	2020	Wrong intervention
Effects of treatment with long-acting muscarinic antagonists and long-acting beta-agonists on lung function improvement in patients with bronchiectasis: An observational study	Lee et al.	2020	Wrong intervention
Clinical characteristics of patients with bronchiectasis and comorbid asthma: The kmbarc registry data	Choi et al.	2020	Wrong study design
Bronchiectasis in India: results from the European Multicentre Bronchiectasis Audit and Research Collaboration (EMBARC) and Respiratory Research Network of India Registry	Dhar et al.	2019	Wrong study design
Airway hyperresponsiveness in bronchiectasis	De et al.	2019	Wrong intervention
Airway clearance in non-cystic fibrosis bronchiectasis: analysis from The United States bronchiectasis research registry	Basavaraj et al.	2019	Wrong study design

Title	Authors/ID	Year	Exclusion reason
The german bronchiectasis registry (PROGNOSIS): Results from 1000 patients	Rademacher et al.	2019	Wrong study design
Mucolytic agents and statins use is associated with a lower risk of acute exacerbations in patients with bronchiectasis-chronic obstructive pulmonary disease overlap	Su et al.	2018	Wrong patient population
Optimization of algorithms of care of patients with infectious extension of bronchiectasis	Antonov et al.	2018	Wrong study design
Mucoactive agents for chronic, non-cystic fibrosis lung disease: A systematic review and meta-analysis	Tarrant et al.	2017	Wrong study design
Hypertonic saline in patients with primary ciliary dyskinesia: On the road to evidencebased treatment for a rare lung disease	Kuehni et al.	2017	Wrong patient population
PROGNOSIS-the German bronchiectasis registry: First results	Mertsch et al.	2017	Wrong study design
Treatment patterns in non-cystic fibrosis bronchiectasis (NCFB): A 2-year assessment post-exacerbation	Germino et al.	2017	Wrong intervention
Management of bronchiectasis in Europe: Data from the European bronchiectasis registry (EMBARC)	Haw et al.	2016	Wrong study design
Phenotype characterization of non cystic fibrosis bronchiectsis in India: Baseline data from Indian bronchiectasis registry	Jalan et al.	2016	No full text and no outcome data in abstract
An analysis of etiology, causal pathogens, imaging patterns, and treatment of Japanese patients with bronchiectasis	Kadowaki et al.	2015	Wrong study design
A comparison of study designs of inhaled agents in Non-Cystic Fibrosis Bronchiectasis (NCFB): Key differences in the phase 3 RESPIRE trials of ciprofloxacin Dry Powder for Inhalation (DPI)	Aksamit et al.	2015	No full text and no outcome data in abstract
Patients in randomized clinical trials of bronchiectasis are only partially representative of clinical practice: A European cohort study	Chalmers et al.	2015	Wrong study design
Mucolytics for bronchiectasis	Wilkinson et al.	2014	Wrong study design
New advances in the therapy of non-cystic fibrosis bronchiectasis	Amorim et al.	2013	Wrong study design
Inhaled mannitol for non-cystic fibrosis bronchiectasis-results of a 12 month, multi-centre, double-blind, controlled study	Bilton et al.	2013	No full text and no outcome data in abstract

Title	Authors/ID	Year	Exclusion reason
Pharmacologic Agents for Mucus Clearance in Bronchiectasis	Nair et al.	2012	Wrong study design
Inhaled mannitol in patients with bronchiectasis: Effect on lung function and health status	Bennoor et al.	2012	No full text and no outcome data in abstract
Nebulised 7% hypertonic saline as an adjunct to airway clearance can be commenced safely in a supervised environment without the need for a formal bronchoconstriction trial	Mills et al.	2012	Wrong comparator
Audit of once daily nebulised hypertonic 6% saline (HTS) in adult non-CF bronchiectasis	Pyne et al.	2010	Wrong study design
Inhaled adrenergics and anticholinergics in obstructive lung disease: Do they enhance mucociliary clearance?	Restrepol et al.	2007	Wrong study design
Inhaled mannitol for the treatment of mucociliary dysfunction in patients with bronchiectasis: Effect on lung function, health status and sputum	Daviskas et al.	2005	Wrong study design
Mannitol, inhaled	Mealy et al.	2005	No full text and no outcome data in abstract
Mucolytics for bronchiectasis	Crockett et al.	2001	Wrong study design
Osmotic stimuli increase clearance of mucus in patients with mucociliary dysfunction	Daviskas et al.	2002	Wrong study design
Nebulisers for bronchiectasis	Currie et al.	1997	Wrong study design
How effective is DNase in managing respiratory diseases other than CF?	Rosenbluth et al.	1999	No full text and no outcome data in abstract
Does a beta2-stimulator really facilitate mucociliary transport in the human lungs in vivo? A study with procaterol	Isawa et al.	1990	Wrong patient population
Clinical trial of ambroxol in 2 different dosage programs in 120 patients with bronchiectasis	Germouty et al.	1988	Wrong patient population
A long term tolerance trial of bromhexine	Mareels et al.	1983	Wrong patient population
The effect of methyl cysteine (Visclair) in respiratory diseases. A pilot study	Sahay et al.	1982	Wrong outcomes
Guaifenesin in chronic hypersecretory bronchopneumopathies: Double-blind clinical trial against placebo	Finiguerra et al.	1982	Article in different language
The use of Duopect as an expectorant antitussive agent	Wojcicki et al.	1975	Wrong patient population
Evaluation and comparison of quality of	ChiCTR2000031817	2020	Ongoing study

Title	Authors/ID	Year	Exclusion reason
life between acetylcysteine tablets and placebo in patients with non cystic fibrosis bronchiectasis			
Efficacy and Safety of An'Ningpai Expectorant in Non-CF Bronchiectasis	NCT04511897	2020	Ongoing study
A Randomized, Controlled Study of Long-term Oral N-acetylcysteine in Patients with Bronchiectasis	ChiCTR-TRC-13003792	2013	Ongoing study
Effects of Traditional Chinese Medicine on Bronchiectasis Patients	NCT03443531	2018	Wrong intervention
Instillation of Gentamicin and Dexamethasone in Bronchiectasis Compared to Conventional Treatment	NCT06209047	2023	Wrong intervention
Benefits of saline solution inhalation prior to respiratory physiotherapy, in patients with bronchiectasis	EUCTR2020-001026-62-ES	2021	Ongoing study
A double-blind, double-simulated, randomized controlled clinical trial on treating phlegm-heat obstructing lung syndrome of stable bronchiectasis with Qingfehuatan Decoction	ChiCTR2400079334	2024	Wrong intervention
Clinical study on modified Cangma Decoction in the treatment of airway mucus hypersecretion in bronchiectasis	ChiCTR2200057265	2022	Wrong intervention
Clinical Observation of Bupi Qingfei Decoction in Treatment of Bronchiectasis Colonized by Pseudomonas aeruginosa	Yuan et al.	2021	Wrong intervention
Is a Single Daily Session Combining HS + Physio Sufficient to Obtain Long-term Clinical Benefits in Bronchiectasis?	NCT04905992	2021	Ongoing study
A randomized, double-blind, placebo-controlled, multicenter clinical study to evaluate the efficacy and safety of inhaled ambroxol hydrochloride solution in improving sputum property of (adult) lower respiratory tract infections	ChiCTR2200066348	2022	Ongoing study
Effect Of N-Acetylcysteine On Exacerbation Of Bronchiectasis	Chowdhury et al.	N/A	No full text and no outcome data in abstract
Mucolytics for bronchiectasis.	Crockett et al.	2000	Wrong study design
The effect of N-acetylcysteine on biofilms: Implications for the treatment of respiratory tract infections	Blasi et al.	2016	Wrong study design
Lung Dispersing, Turbid Descending and Gut Clearing Decoction for Bronchiectasis	Guangzhou Institute of Respiratory	2023	Ongoing study
Study of Safety, Tolerability, Pharmacokinetics and Pharmacodynamics of QBW251 in Subjects With Bronchiectasis	Novartis	2023	No full text and no outcome data in abstract

Title	Authors/ID	Year	Exclusion reason
Phase 2a, 28-day Investigational Use Study of ARINA-1 in Non-CF Bronchiectasis With Excess Mucus and Cough	Renovion Inc	2024	Ongoing study
Efficacy and Safety of An'Ningpai Expectorant in Non-CF Bronchiectasis	Shanghai Pulmonary Hospital Shanghai China	2022	Ongoing study
Safety, Tolerability and Efficacy of S-1226 in Cystic Fibrosis and Non CF Bronchiectasis	SolAeroMed	2022	Wrong patient population
Hypertonic Saline in NCFB	University of North Carolina et al.	2025	Ongoing study
A Clinical Trial to Compare the Efficacy and Safety of 1-week Treatment of Intravenous N-acetylcysteine (NAC) 600 mg Twice Daily, Ambroxol Hydrochloride 30 mg Twice Daily and Placebo as Expectorant Therapies in Adult Chinese Patients With Respiratory Tract Diseases and Abnormal Mucus Secretions	Zambon Sp A.	2021	Wrong patient population
Use of nebulised saline and nebulised terbutaline as an adjunct to chest physiotherapy.	Sutton et al.	1988	Wrong intervention
Inhaled mannitol improves lung function assessed by forced oscillation in a placebo controlled trial in patients with bronchiectasis	Goldman et al.	N/A	No full text and no outcome data in abstract
Impact of saline solution's nebulisation on the biophysical properties of sputum in bronchiectasis	Alcaraz-Serrano et al.	2024	No full text and no outcome data in abstract
Brensocatib in non-cystic fibrosis bronchiectasis: ASPEN protocol and baseline characteristics	Chalmers et al.	2024	Wrong intervention
Psychometric Validation and Determination of the Minimal Clinically Important Difference for Bronchiectasis Health Questionnaire in Adults with Bronchiectasis	Xu et al.	2024	Wrong intervention
Comparative effectiveness and safety of inhaled corticosteroid plus long-acting beta<ovid:inf>2</ovid:inf>-agonist fixed-dose combinations vs. long-acting muscarinic antagonist in bronchiectasis	Su et al.	2024	Wrong intervention
Using Continuous Cough Monitoring to Assess Bronchiectasis Therapy	Griffith et al.	2024	Wrong study design
Novel Anti-inflammatory and Immunomodulatory Effects of the Dipeptidyl Peptidase-1 Inhibitor Brensocatib: A Post-hoc Analysis of the WILLOW Trial	Johnson et al.	2024	Wrong intervention
What is the optimal dose of N-	Oscullo et al.	2025	Wrong study

Title	Authors/ID	Year	Exclusion reason
acetylcysteine in adult patients with bronchiectasis?-data from the RIBRON registry			design
Safety and efficacy of inhalable ambroxol hydrochloride aerosol for adult patients with respiratory diseases: An open-label, single-Arm, multicentre study	Ma et al.	2025	Wrong patient population
A Multicenter, Randomized, Double-blind, Placebo-controlled Phase II Clinical Study to Evaluate the Efficacy, Safety, Pharmacokinetics, and Pharmacodynamics of Inhaled H057 in the Treatment of Acute Exacerbations of Bronchiectasis	NCT06958861	2025	Ongoing study
Efficacy of acetylcysteine nebulizer for improvement of symptoms in bronchiectasis patients: a randomized controlled trial	Kijlertsuphasri et al.	2025	Ongoing study
A confirmatory clinical trial of inhaled bromhexine hydrochloride solution for adult expectorant therapy	Author not found	2025	Ongoing study

Ongoing studies

Table S6 - List of articles excluded as 'ongoing studies'.

	Record title	Associated investigators/institutions	Registration Number	Year
1	The effect of N-acetylcysteine in patients with non-cystic fibrosis bronchiectasis (NINCFB): study protocol for a multicentre, double-blind, randomised, placebo-controlled trial.	Liao *et al.*	ChiCTR2000031817	2022
2	Evaluation and comparison of quality of life between acetylcysteine tablets and placebo in patients with non cystic fibrosis bronchiectasis	Liao *et al.* (additional record of same study)	ChiCTR2000031817	2020
3	Efficacy and Safety of An'Ningpai Expectorant in Non-CF Bronchiectasis	Yu *et al.*	NCT04511897	2020
4	A Randomized, Controlled Study of Long-term Oral N-acetylcysteine in Patients with Bronchiectasis	Xu *et al.*	ChiCTR-TRC-13003792	2013
5	Benefits of saline solution inhalation prior to respiratory physiotherapy, in patients with bronchiectasis	Trueta *et al.*	2020-001026-62 (EU)	2021
6	Is a Single Daily Session Combining HS + Physio Sufficient to Obtain Long-term Clinical Benefits in Bronchiectasis?	Herrero-Cortina *et al.*	NCT04905992	2021
7	A randomized, double-blind, placebo-controlled, multicenter clinical study to evaluate the efficacy and safety of inhaled ambroxol hydrochloride solution in improving sputum property of (adult) lower respiratory tract infections	Guan *et al.*	ChiCTR2200066348	2022
8	Lung Dispersing, Turbid Descending and Gut Clearing Decoction for Bronchiectasis (LUNG-CLEAR)	Chen *et al.*	NCT03177889	2023
9	Phase 2a, 28-day Investigational Use Study of ARINA-1 in Non-CF Bronchiectasis With Excess Mucus and Cough	Daley *et al.*	NCT05495243	2024
10	Efficacy and Safety of An'Ningpai Expectorant in Non-CF Bronchiectasis	Xu *et al.*	NCT04511897	2022

	Record title	Associated investigators/institutions	Registration Number	Year
11	Hypertonic Saline in NCFB	University of North Carolina, Chapel Hill	NCT06242795	2025
12	A Multicenter, Randomized, Double-blind, Placebo-controlled Phase II Clinical Study to Evaluate the Efficacy, Safety, Pharmacokinetics, and Pharmacodynamics of Inhaled H057 in the Treatment of Acute Exacerbations of Bronchiectasis	Shanghai Huilun Pharmaceutical Co., Ltd.	NCT06958861	2025
13	Efficacy of acetylcysteine nebulizer for improvement of symptoms in bronchiectasis patients: a randomized controlled trial	Kijlertsuphasri et al.	TCTR20250707006	2025
14	A confirmatory clinical trial of inhaled bromhexine hydrochloride solution for adult expectorant therapy	Shenzhen People's Hospital	ChiCTR2500097308	2025

Risk of Bias

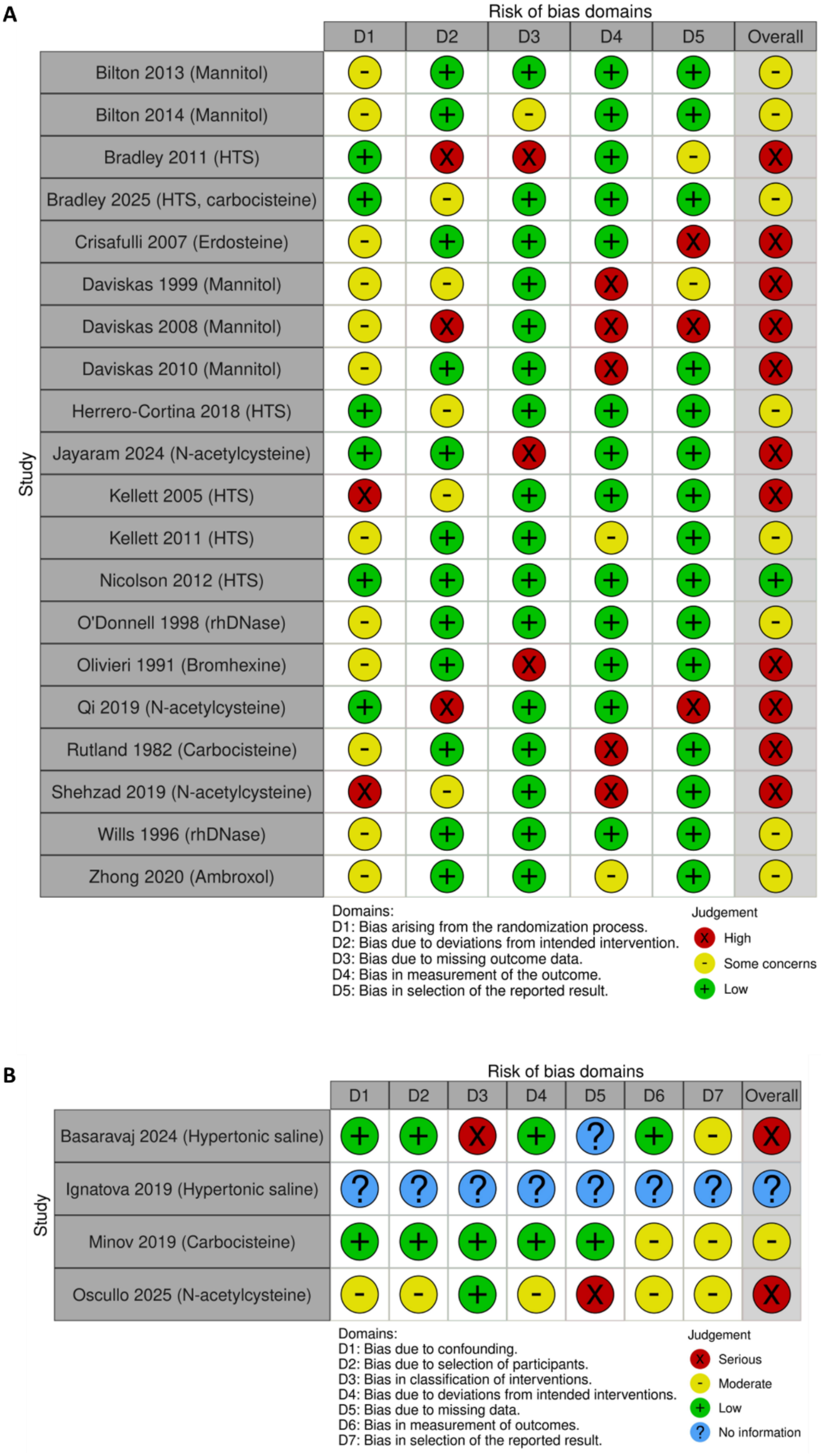

Figure S1 - Risk of bias judgements for randomised (A) and non-randomised studies (B).

Additional Data

Exacerbation Duration

Meta-analysis of two randomised trials (501 patients) showed no significant effect of mucoactive treatment on exacerbation duration (Figure S2). The overall mean difference was an increase of 2.56 days per exacerbation for patients allocated a mucoactive agent (95% CI: –11.39, 16.51; p=0.72). Heterogeneity was moderate (I^2=69.3%).

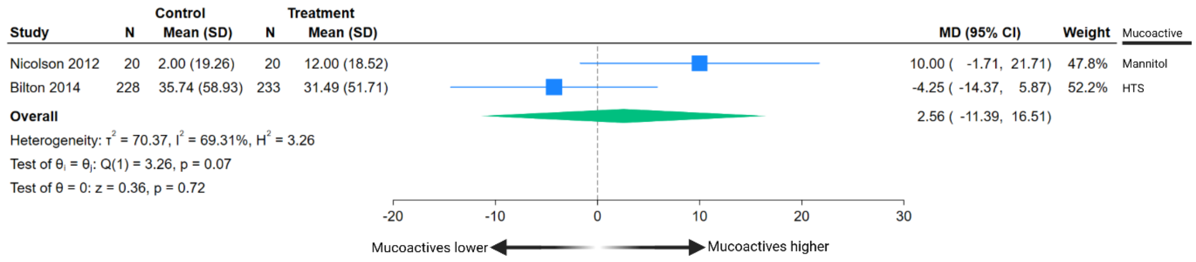

Figure S2 - Meta-analysis of exacerbation duration (days) between mucoactive treatment and control groups. *Mean differences in exacerbation duration from two studies were analysed. HTS – hypertonic saline.*

Lung Function

Forced Expiratory Volume in 1 second (FEV_1)

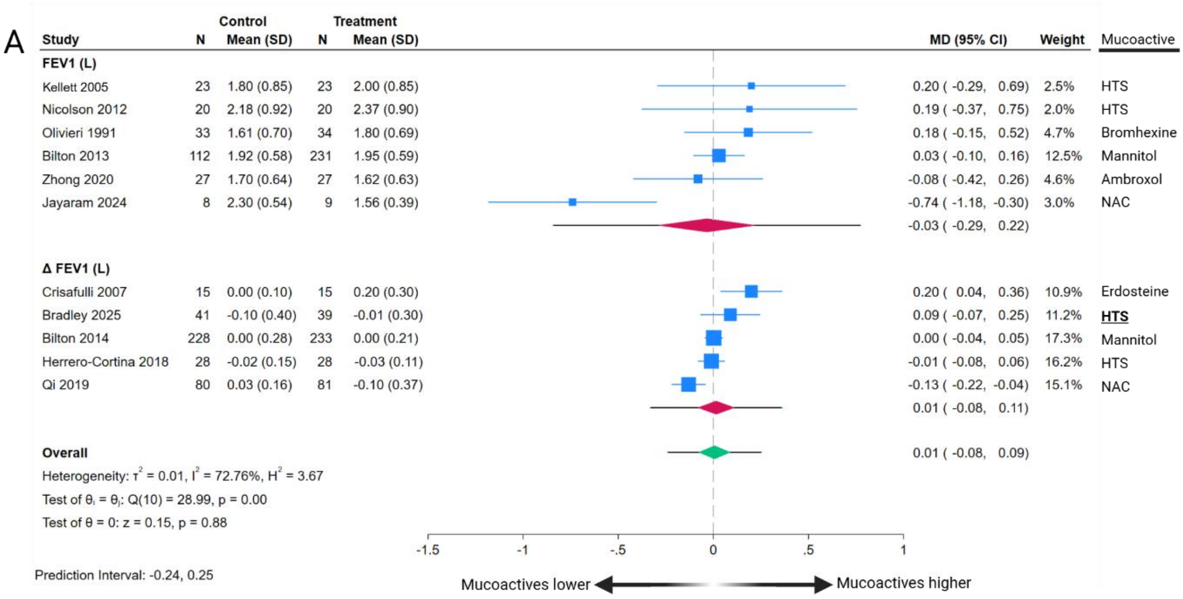

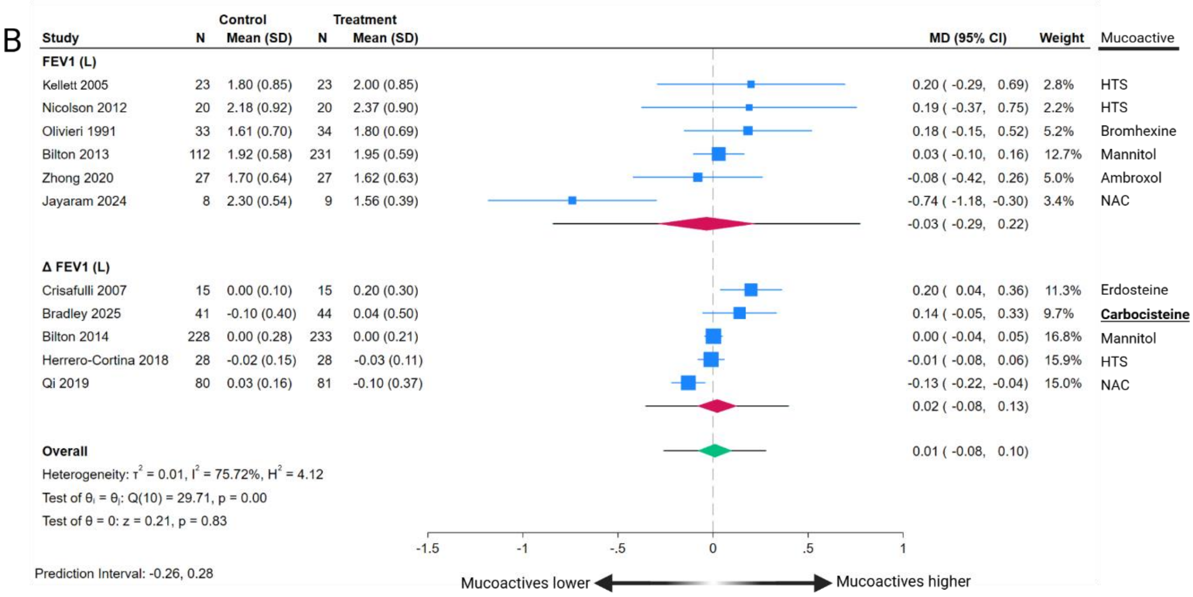

Figure S3 -- Meta-analysis of mean differences in FEV_1 (L) between mucoactive treatment and control groups. *(A) Mean differences and changes in FEV_1 (L) from eleven studies were analysed. HTS – hypertonic saline; NAC – N-acetylcysteine. (B) Sensitivity analysis, including Bradley 2025 carbocisteine data.*

Forced Vital Capacity (FVC)

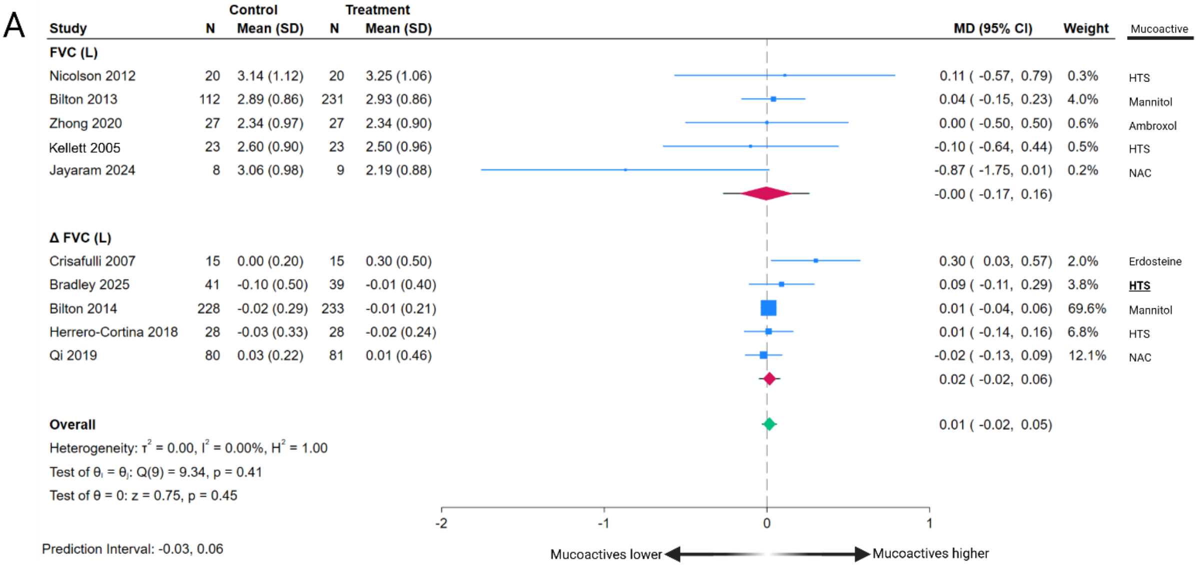

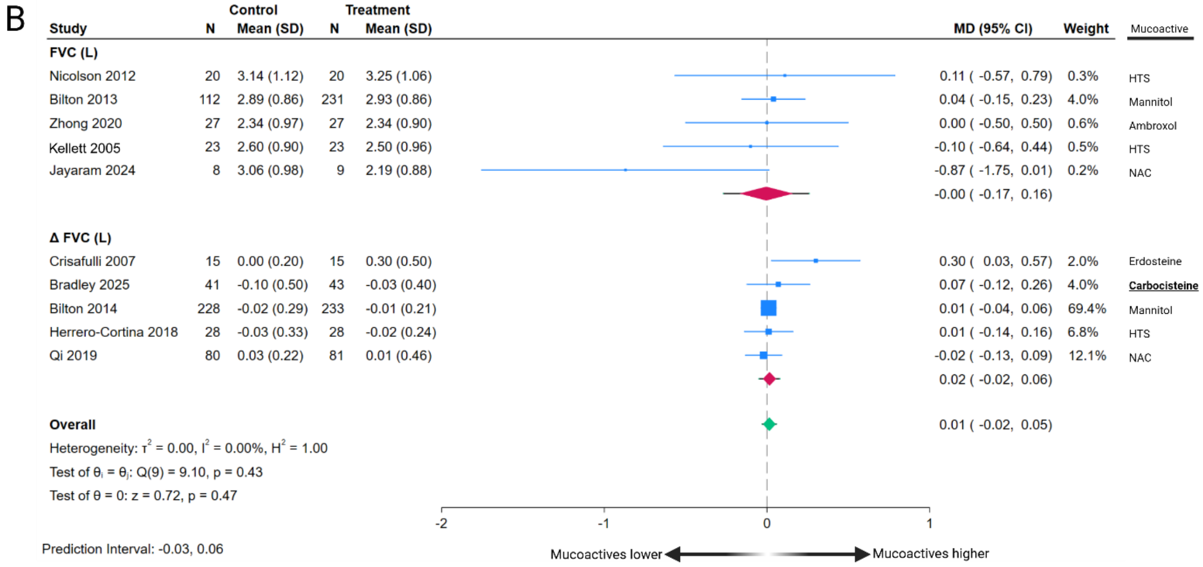

Figure S4 – Meta-analysis of FVC (L) between mucoactive treatment and control groups. *Mean differences and changes in FVC from nine studies were analysed. HTS – hypertonic saline; NAC – N-acetylcysteine. (B) Sensitivity analysis, including Bradley 2025 carbocisteine data.*

FVC Percentage Predicted

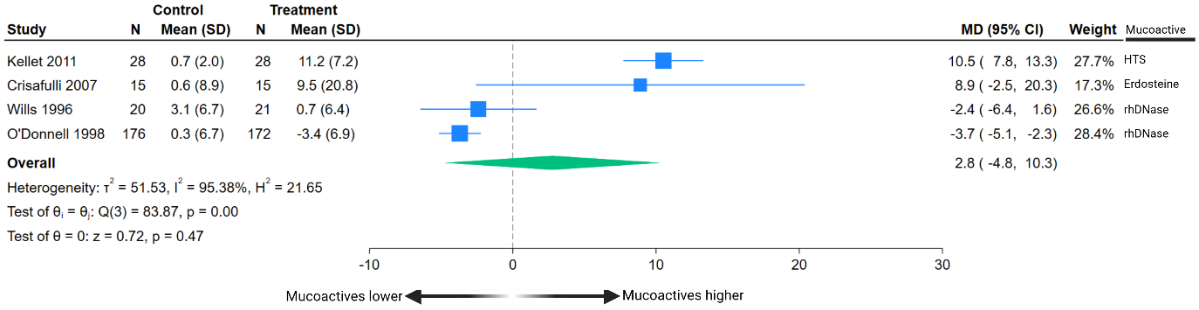

Figure S5 - Meta-analysis of mean differences in FVC percentage predicted between mucoactive treatment and control groups. *Mean differences in FVC% from four studies were analysed. HTS – hypertonic saline.*

FEV_1/FVC Ratio

Meta-analysis of three randomised trials (205 patients) showed no significant effect of mucoactive treatment on FEV1/FVC ratio (Figure S6). The mean difference was –0.9% (95% CI: –2.7, 0.9; p=0.32; I^2=0.0%).

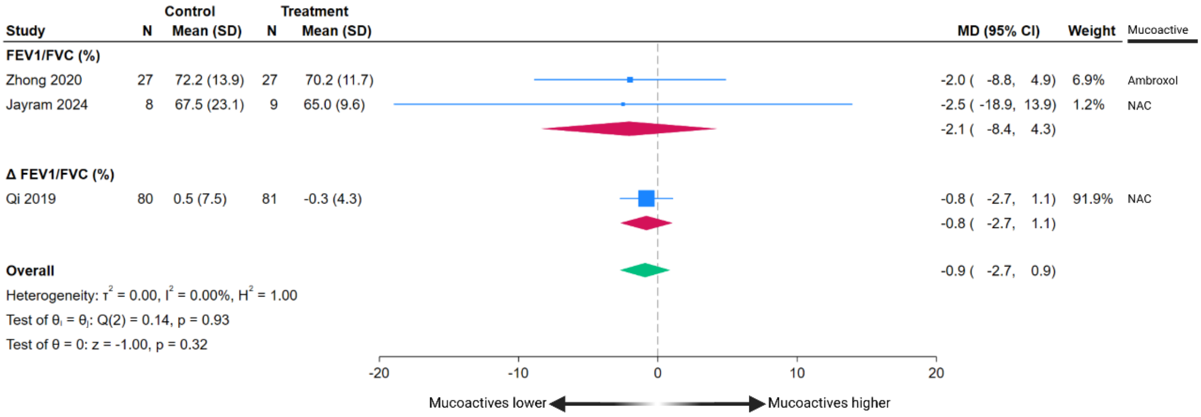

Figure S6 - Meta-analysis of the FEV_1/FVC ratio between mucoactive treatment and control groups. *Mean differences and changes in FEV_1/FVC from three studies were analysed. NAC – N-acetylcysteine.*

Sputum characteristics & microbiology

Sputum Volume

Meta-analysis of two randomised trials (188 patients) showed no significant effect of mucoactive treatment on 24-hour sputum volume (Figure S7). The mean difference was –6.1 mL (95% CI: –15.8, 3.5; p=0.21) but heterogeneity was substantial (I^2=84.4%).

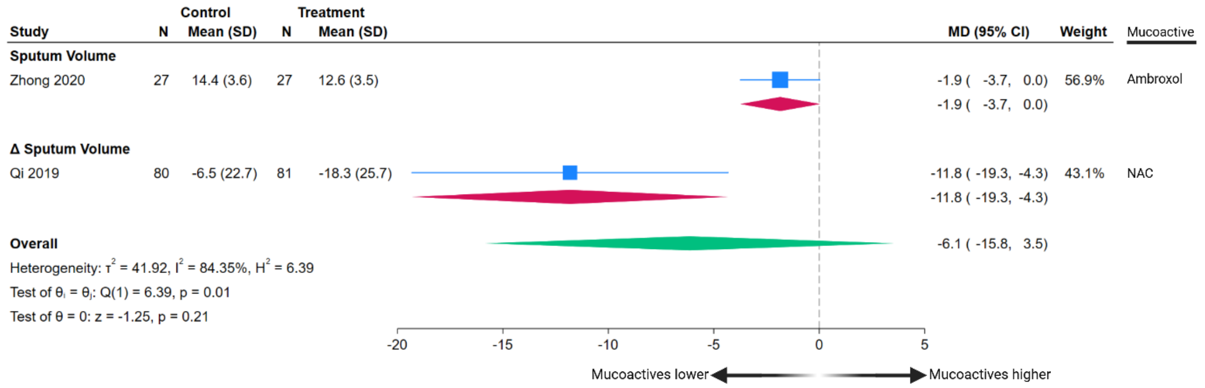

Figure S7 – Meta-analysis of 24-hour sputum volume (mL) between mucoactive treatment and control groups. *Mean differences and changes in sputum volume from two studies were analysed.; NAC – N-acetylcysteine.*

Sputum Weight

Meta-analysis of two randomised trials (both evaluating expectorant mannitol; 804 patients) showed mucoactive treatment significantly increased 24-hour sputum weight compared to control (Figure S8) [2,3]. The mean difference was 3.4 g (95% CI: 1.7, 5.1; p<0.001; I^2=0.0%).

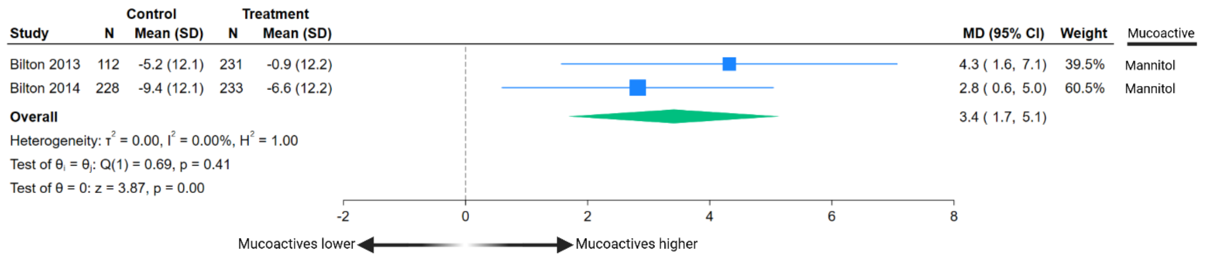

Figure S8 - Meta-analysis of 24-hour sputum weight (g) between mannitol treatment and control groups. *Mean differences in sputum weight from two studies were analysed, showing a significant increase with mannitol (p<0.001).*

Pseudomonas aeruginosa status

Meta-analysis of two observational cohort studies (1405 patients) showed no significant effect of mucoactive treatment on the odds of positive *Pseudomonas aeruginosa* status (Figure S9). The overall odds ratio was 1.13 (95% CI: 0.84, 1.52; p=0.43; 0.0%).

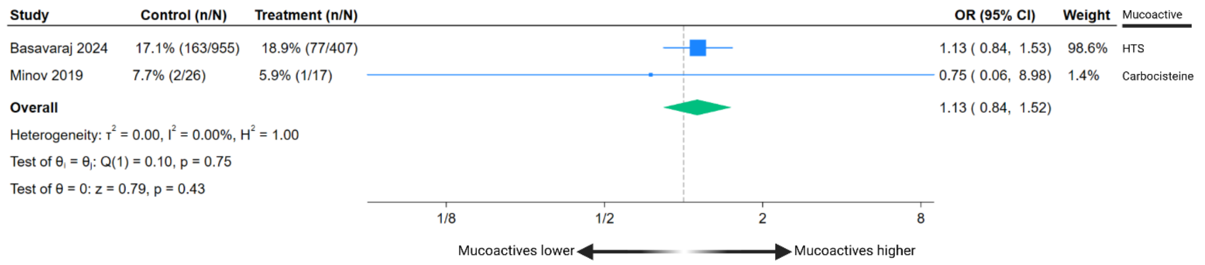

Figure S9 - Meta-analysis of Pseudomonas aeruginosa status in bronchiectasis patients following mucoactive treatment versus control. *Proportions of patients positive for Pseudomonas aeruginosa from two studies were analysed. HTS – hypertonic saline.*

Safety

Hospital Admissions

Meta-analysis of two randomised trials (81 patients) showed no significant effect of mucoactive treatment on the odds of hospital admission (Figure S10). The overall odds ratio was 0.40 (95% CI: 0.05, 3.06; p=0.38; I^2=0.0%).

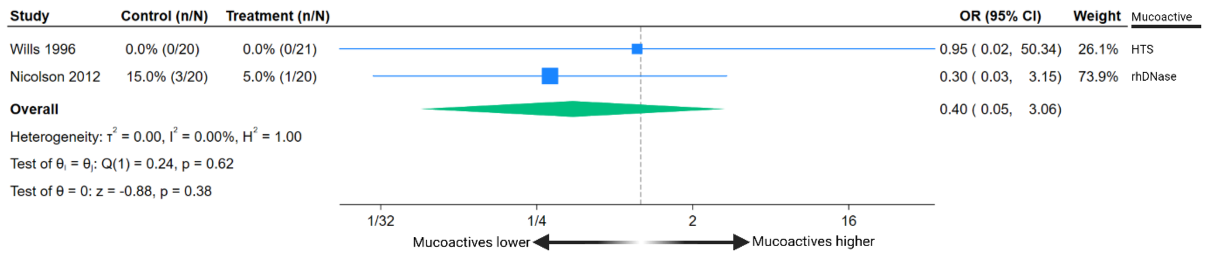

Figure S10 – Meta-analysis of hospital admissions in bronchiectasis patients following mucoactive treatment versus control. *Proportions of patients with hospital admissions from two studies were analysed. HTS – hypertonic saline.*

Quality of Life

Total LCQ Scores

Meta-analysis of three randomised trials (398 patients) showed no significant effect of mucoactive treatment on LCQ scores (Figure S11). The mean difference was 0.04 (95% CI: –0.37, 0.46; p=0.85; I^2=0.0%).

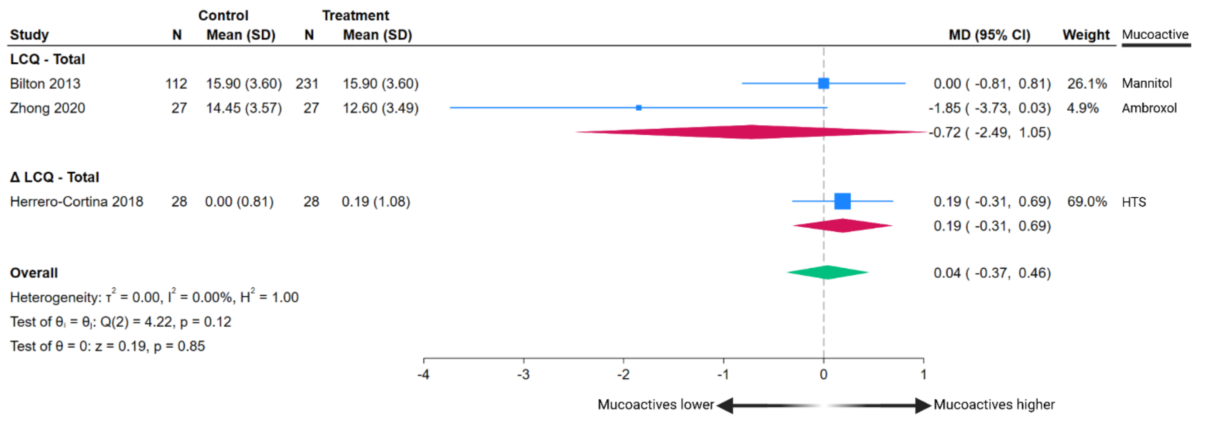

Figure S11 – Meta-analysis of Leicester Cough Questionnaire (LCQ) scores between mucoactive treatment and control groups in bronchiectasis. *Total LCQ scores range from 3 to 21, with lower scores indicating worse cough and decreased quality of life. Scores from three studies were analysed. HTS – hypertonic saline.*

Exercise Capacity

Meta-analysis of two randomised trials (57 patients) showed no significant effect of mucoactive treatment on exercise capacity as measured by 6MWT distance (Figure S12). The mean difference was –8.1 m (95% CI: –41, 24.8; p=0.63; I^2=0.0%).

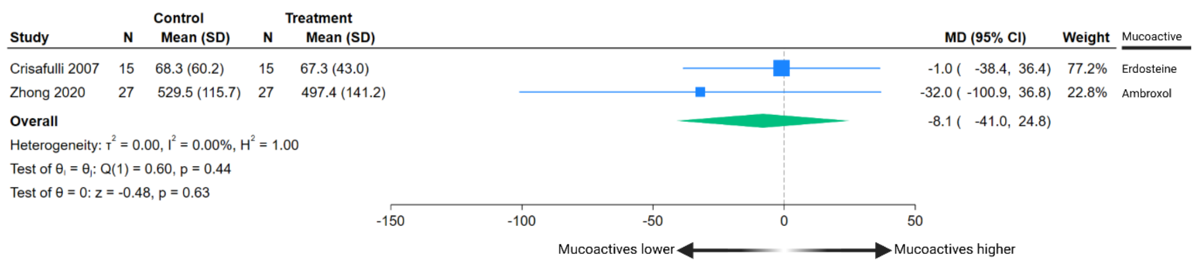

Figure S12 – Meta-analysis of exercise capacity between mucoactive treatment and control groups in bronchiectasis, as measured by 6-Minute Walk Test distance in metres. *Distances from two studies were analysed.*

GRADE findings

Evidence profile

Table S7 - GRADE evidence profile for all outcomes. Question: Mucoactives compared to Control for Adults with Non-CF Bronchiectasis.

Certainty assessment							№ of patients		Effect		Certainty	Importance
№ of studies	Study design	Risk of bias	Inconsistency	Indirectness	Imprecision	Other considerations	Mucoactives	Control	Relative (95% CI)	Absolute (95% CI)		
**Mean difference in FEV1 (L) (follow-up: range 1 weeks to 52; assessed with: Mean (SD))**												
11	randomised trials	serious[a,b]	serious[c]	not serious	serious[b]	none	740	615	-	MD **0.01 L** (0.08 lower to 0.09 higher)	⨁◯◯◯ Very low[a,b,c]	IMPORTANT
**Mean difference in Percentage Predicted FEV1 (%) (follow-up: range 1 days to 52 weeks; assessed with: Mean (SD))**												
9	randomised trials	serious[a]	serious[c]	not serious	not serious	publication bias strongly suspected[d]	417	419	-	MD **3.3 % higher** (0.2 higher to 6.4 higher)	⨁◯◯◯ Very low[a,c,d]	IMPORTANT
**Mean difference in FVC (L) (follow-up: range 1 weeks to 52 weeks; assessed with: Mean (SD))**												
10	randomised trials	serious[a]	not serious	not serious	serious[b]	none	706	582	-	MD **0.01 L higher** (0.02 lower to 0.05 higher)	⨁⨁◯◯ Low[a,b]	IMPORTANT
**Mean difference in Percentage Predicted FVC (%) (follow-up: range 2 weeks to 24 weeks; assessed with: Mean (SD))**												
4	randomised trials	not serious	serious[c]	not serious	serious[b]	publication bias strongly suspected[e]	236	239	-	MD **2.8 % higher** (4.8 lower to 10.3 higher)	⨁◯◯◯ Very low[b,c,e]	IMPORTANT
**Mean difference in FEV1/FVC ratio (%) (follow-up: range 6 weeks to 52 weeks; assessed with: Mean (SD))**												

Certainty assessment							№ of patients		Effect		Certainty	Importance
№ of studies	Study design	Risk of bias	Inconsistency	Indirectness	Imprecision	Other considerations	Mucoactives	Control	Relative (95% CI)	Absolute (95% CI)		
3	randomised trials	serious[a]	not serious	not serious	serious[b]	publication bias strongly suspected[e]	117	115	-	MD **0.9 % lower** (2.7 lower to 0.9 higher)	⨁◯◯◯ Very low[a,b,e]	IMPORTANT
**Pulmonary Exacerbation Incidence (follow-up: range 12 weeks to 52 weeks; assessed with: Mean (SD) n/year)**												
9	randomised trials[f]	not serious	serious[c]	not serious	serious[b]	publication bias strongly suspected[e]	1029	2755	-	MD **0.56 exacerbations/year lower** (1.30 lower to 0.19 higher)	⨁◯◯◯ Very low[b,c,e]	CRITICAL
**Pulmonary Exacerbation Duration (follow-up: range 12 weeks to 52 weeks; assessed with: Mean (SD) n of days)**												
2	randomised trials	not serious	serious[c]	not serious	serious[b]	publication bias strongly suspected[e]	253	248	-	MD **2.56 days higher** (11.39 lower to 16.51 higher)	⨁◯◯◯ Very low[b,c,e]	CRITICAL
**Shortness of Breath - not measured**												
-	-	-	-	-	-	-	-	-	-	-	-	CRITICAL
**Quality of Life (follow-up: range 12 weeks to 52 weeks; assessed with: Mean (SD) Overall SGRQ Score)**												
4	randomised trials	not serious	not serious	not serious	serious[b]	publication bias strongly suspected[e]	544	421	-	MD **1.56 lower** (3.25 lower to 0.14 higher)	⨁⨁◯◯ Low[b,e]	CRITICAL
**Cough (follow-up: range 3 weeks to 12 weeks; assessed with: Mean (SD) Total LCQ score)**												

Certainty assessment							№ of patients		Effect		Certainty	Importance
№ of studies	Study design	Risk of bias	Inconsistency	Indirectness	Imprecision	Other considerations	Mucoactives	Control	Relative (95% CI)	Absolute (95% CI)		
3	randomised trials	not serious	not serious	not serious	serious[b]	publication bias strongly suspected[e]	286	167	-	MD **0.04 higher** (0.37 lower to 0.46 higher)	⨁⨁◯◯ Low[b,e]	CRITICAL
**Exercise Capacity (follow-up: range 2 weeks to 12 weeks; assessed with: Mean (SD) 6MWT distance)**												
2	randomised trials	serious[a]	not serious	not serious	serious[b]	publication bias strongly suspected[e]	42	42	-	MD **8.1 m lower** (41 lower to 24.8 higher)	⨁◯◯◯ Very low[a,b,e]	CRITICAL
**Sputum Volume (assessed with: 24h volume)**												
2	randomised trials	serious[a]	serious[c]	not serious	serious[b]	publication bias strongly suspected[e]	108	107	-	MD **6.1 ml lower** (15.8 lower to 3.5 higher)	⨁◯◯◯ Very low[a,b,c,e]	IMPORTANT
**Sputum Weight (follow-up: range 12 weeks to 52 weeks; assessed with: 24h weight)**												
2	randomised trials	serious[a]	not serious	not serious	not serious	publication bias strongly suspected[e]	464	340	-	MD **3.4 g higher** (1.7 higher to 5.1 higher)	⨁⨁◯◯ Low[a,e]	IMPORTANT
**Sputum Microbiology (assessed with: P. Aeruginosa status)**												
2	non-randomised studies	serious[a]	not serious	not serious	serious[b]	publication bias strongly suspected[e]	78/424 (18.4%)	165/981 (16.8%)	**OR 1.13** (0.84 to 1.52)	**2 more per 100** (from 2 fewer to 7 more)	⨁◯◯◯ Very low[a,b,e]	CRITICAL
**Patients' Perception of Health - not measured**												
-	-	-	-	-	-	-	-	-	-	-	-	IMPORTANT

Certainty assessment							№ of patients		Effect		Certainty	Importance
№ of studies	Study design	Risk of bias	Inconsistency	Indirectness	Imprecision	Other considerations	Mucoactives	Control	Relative (95% CI)	Absolute (95% CI)		
**Serious Adverse Events (follow-up: range 6 weeks to 52 weeks)**												
4	randomised trials	serious[a]	not serious	not serious	serious[b]	publication bias strongly suspected[e]	61/546 (11.2%)	66/420 (15.7%)	**OR 0.79** (0.54 to 1.16)	**3 fewer per 100** (from 7 fewer to 3 more)	⨁◯◯◯ Very low[a,b,e]	CRITICAL
**Hospital Admissions (follow-up: range 4 weeks to 52 weeks)**												
2	randomised trials	not serious	not serious	not serious	serious[b]	publication bias strongly suspected[e]	1/41 (2.4%)	3/40 (7.5%)	**OR 0.40** (0.05 to 3.06)	**92 fewer per 1,000** (from 156 fewer to 212 more)	⨁⨁◯◯ Low[b,e]	CRITICAL

CI: confidence interval; **MD:** mean difference; **OR:** odds ratio

Explanations

a. Risk of bias was deemed Serious as the majority of trials had high risk of bias in at least one domain.

b. Imprecision was deemed serious due to the width of the CIs, inclusion of the line of no effect or uncertainty over clinical significance of effect.

c. Inconsistency was deemed Serious due to high heterogeneity.

d. There is a strong suspicion of publication bias in the evidence, as indicated by the asymmetry observed in the funnel plot and a significant p-value from the Egger test. This suggests that smaller studies with non-significant or negative results may be underrepresented in the analysis, potentially leading to an overestimation of the treatment effect. As a result, the confidence in the estimated effect size is reduced.

e. Although there is a suspicion of publication bias, the small number of studies (fewer than 9) limits the reliability of the funnel plot and Egger test in detecting such bias. This limitation makes it challenging to conclusively determine the presence of publication bias, but the potential for bias cannot be ruled out, which may affect the confidence in the estimated effect size.

f. Two observational studies (Minov 2019 and Oscullo 2025) included with RCTs.

Summary of findings: exacerbations and lung function

Table S8 - GRADE summary of findings: exacerbations and lung function outcomes.

Outcomes	Anticipated absolute effects[*] (95% CI)		Relative effect (95% CI)	№ of participants (studies)	Certainty of the evidence (GRADE)	Comments
	**Risk with Control**	**Risk with Mucoactives**				
Pulmonary Exacerbation Incidence assessed with: Mean (SD) n/year follow-up: range 12 weeks to 52 weeks		MD **0.56 exacerbations/year lower** (1.30 lower to 0.19 higher)	-	3729 (9 RCTs)[a]	⨁◯◯◯ Very low[b,c,d]	Mucoactives may result in little to no difference in pulmonary Exacerbation Incidence.
Pulmonary Exacerbation Duration assessed with: Mean (SD) n of days follow-up: range 12 weeks to 52 weeks		MD **2.56 days higher** (11.39 lower to 16.51 higher)	-	501 (2 RCTs)	⨁◯◯◯ Very low[b,c,d]	Mucoactives may reduce/have little to no effect on pulmonary Exacerbation Duration but the evidence is very uncertain.
Mean difference in FEV1 (L) assessed with: Mean (SD) follow-up: range 1 weeks to 52		MD **0.01 L** (0.08 lower to 0.09 higher)	-	1277 (11 RCTs)	⨁◯◯◯ Very low[b,c,e]	Mucoactives may have little to no effect on mean difference in FEV1 (L) but the evidence is very uncertain.
Mean difference in Percentage Predicted FEV1 (%) assessed with: Mean (SD) follow-up: range 1 days to 52 weeks		MD **3.3 % higher** (0.2 higher to 6.4 higher)	-	767 (9 RCTs)	⨁◯◯◯ Very low[b,e,f]	The evidence suggests mucoactives results in a slight increase in mean difference in Percentage Predicted FEV1 (%) but the evidence is very uncertain.

Outcomes	Anticipated absolute effects* (95% CI)		Relative effect (95% CI)	№ of participants (studies)	Certainty of the evidence (GRADE)	Comments
	Risk with Control	Risk with Mucoactives				
Mean difference in FVC (L) assessed with: Mean (SD) follow-up: range 1 weeks to 52 weeks		MD **0.01 L higher** (0.02 lower to 0.05 higher)	-	1210 (10 RCTs)	⨁⨁◯◯ Low[c,e]	Mucoactives may result in little to no difference in mean difference in FVC (L).
Mean difference in Percentage Predicted FVC (%) assessed with: Mean (SD) follow-up: range 2 weeks to 24 weeks		MD **2.8 % higher** (4.8 lower to 10.3 higher)	-	477 (4 RCTs)	⨁◯◯◯ Very low[b,c,d]	Mucoactives may result in little to no difference in mean difference in Percentage Predicted FVC (%).
Mean difference in FEV1/FVC ratio (%) assessed with: Mean (SD) follow-up: range 6 weeks to 52 weeks		MD **0.9 % lower** (2.7 lower to 0.9 higher)	-	205 (3 RCTs)	⨁◯◯◯ Very low[c,d,e]	Mucoactives may reduce/have little to no effect on mean difference in FEV1/FVC ratio (%) but the evidence is very uncertain.

***The risk in the intervention group** (and its 95% confidence interval) is based on the assumed risk in the comparison group and the **relative effect** of the intervention (and its 95% CI).

CI: confidence interval; **MD:** mean difference; **OR:** odds ratio

GRADE Working Group grades of evidence
High certainty: we are very confident that the true effect lies close to that of the estimate of the effect.
Moderate certainty: we are moderately confident in the effect estimate: the true effect is likely to be close to the estimate of the effect, but there is a possibility that it is substantially different.
Low certainty: our confidence in the effect estimate is limited: the true effect may be substantially different from the estimate of the effect.
Very low certainty: we have very little confidence in the effect estimate: the true effect is likely to be substantially different from the estimate of effect.

Explanations

a. Two observational studies (Minov 2019 and Oscullo 2025) included with RCTs.

b. Inconsistency was deemed Serious due to high heterogeneity.

c. Imprecision was deemed serious due to the width of the CIs, inclusion of the line of no effect or uncertainty over clinical significance of effect.

d. Although there is a suspicion of publication bias, the small number of studies (fewer than 9) limits the reliability of the funnel plot and Egger test in detecting such bias. This limitation makes it challenging to conclusively determine the presence of publication bias, but the potential for bias cannot be ruled out, which may affect the confidence in the estimated effect size.

e. Risk of bias was deemed Serious as the majority of trials had high risk of bias in at least one domain.

f. There is a strong suspicion of publication bias in the evidence, as indicated by the asymmetry observed in the funnel plot and a significant p-value from the Egger test. This suggests that smaller studies with non-significant or negative results may be underrepresented in the analysis, potentially leading to an overestimation of the treatment effect. As a result, the confidence in the estimated effect size is reduced.

Summary of findings: symptoms and sputum characteristics

Table S9 - GRADE summary of findings: symptoms and sputum characteristics.

Outcomes	Anticipated absolute effects* (95% CI)		Relative effect (95% CI)	№ of participants (studies)	Certainty of the evidence (GRADE)	Comments
	Risk with Control	Risk with Mucoactives				
Shortness of Breath - not measured	-	-	-	-	-	
Quality of Life (QoL) assessed with: Mean (SD) Overall SGRQ Score follow-up: range 12 weeks to 52 weeks		MD **1.56 lower** (3.25 lower to 0.14 higher)	-	937 (4 RCTs)	⨁⨁◯◯ Low[b,c]	Mucoactives may result in little to no difference in quality of Life .
Cough assessed with: Mean (SD) Total LCQ score follow-up: range 3 weeks to 12 weeks		MD **0.04 higher** (0.37 lower to 0.46 higher)	-	398 (3 RCTs)	⨁⨁◯◯ Low[b,c]	Mucoactives may result in little to no difference in cough.
Exercise Capacity assessed with: Mean (SD) 6MWT distance follow-up: range 2 weeks to 12 weeks		MD **8.1 m lower** (41 lower to 24.8 higher)	-	57 (2 RCTs)	⨁◯◯◯ Very low[b,c,d]	Mucoactives may have little to no effect on exercise Capacity but the evidence is very uncertain.
Sputum Volume assessed with: 24h volume		MD **6.1 ml lower** (15.8 lower to 3.5 higher)	-	188 (2 RCTs)	⨁◯◯◯ Very low[a,b,c,d]	Mucoactives may have little to no effect on sputum Volume but the evidence is very uncertain.

Outcomes	Anticipated absolute effects* (95% CI)		Relative effect (95% CI)	№ of participants (studies)	Certainty of the evidence (GRADE)	Comments
	Risk with Control	Risk with Mucoactives				
Sputum Weight assessed with: 24h weight follow-up: range 12 weeks to 52 weeks		MD **3.4 g higher** (1.7 higher to 5.1 higher)	-	804 (2 RCTs)	⊕⊕◯◯ Low[c,d]	The evidence suggests mucoactives results in a slight increase in sputum Weight.
Sputum Microbiology assessed with: P. Aeruginosa status	17 per 100	**19 per 100** (15 to 24)	**OR 1.13** (0.84 to 1.52)	1405 (2 non-randomised studies)	⊕◯◯◯ Very low[b,c,d]	Mucoactives may increase/have little to no effect on sputum Microbiology but the evidence is very uncertain.

***The risk in the intervention group** (and its 95% confidence interval) is based on the assumed risk in the comparison group and the **relative effect** of the intervention (and its 95% CI).

CI: confidence interval; **MD:** mean difference; **OR:** odds ratio

GRADE Working Group grades of evidence
High certainty: we are very confident that the true effect lies close to that of the estimate of the effect.
Moderate certainty: we are moderately confident in the effect estimate: the true effect is likely to be close to the estimate of the effect, but there is a possibility that it is substantially different.
Low certainty: our confidence in the effect estimate is limited: the true effect may be substantially different from the estimate of the effect.
Very low certainty: we have very little confidence in the effect estimate: the true effect is likely to be substantially different from the estimate of effect.

Explanations

a. Inconsistency was deemed Serious due to high heterogeneity.

b. Imprecision was deemed serious due to the width of the CIs, inclusion of the line of no effect or uncertainty over clinical significance of effect.

c. Although there is a suspicion of publication bias, the small number of studies (fewer than 9) limits the reliability of the funnel plot and Egger test in detecting such bias. This limitation makes it challenging to conclusively determine the presence of publication bias, but the potential for bias cannot be ruled out, which may affect the confidence in the estimated effect size.

d. Risk of bias was deemed Serious as the majority of trials had high risk of bias in at least one domain.

Summary of findings: safety

Table S10 - GRADE summary of findings: safety.

Outcomes	Anticipated absolute effects* (95% CI)		Relative effect (95% CI)	№ of participants (studies)	Certainty of the evidence (GRADE)	Comments
	**Risk with Control**	**Risk with Mucoactives**				
Patients' Perception of Health - not measured	-	-	-	-	-	
Serious Adverse Events (SAEs) follow-up: range 6 weeks to 52 weeks	16 per 100	**14 per 100** (10 to 19)	**OR 0.79** (0.54 to 1.16)	966 (4 RCTs)	⨁◯◯◯ Very low[a,b,c]	Mucoactives may reduce/have little to no effect on serious Adverse Events but the evidence is very uncertain.
Hospital Admissions follow-up: range 4 weeks to 52 weeks	166 per 1,000	**74 per 1,000** (10 to 378)	**OR 0.40** (0.05 to 3.06)	81 (2 RCTs)	⨁⨁◯◯ Low[a,b]	Mucoactives may result in little to no difference in hospital Admissions.

***The risk in the intervention group** (and its 95% confidence interval) is based on the assumed risk in the comparison group and the **relative effect** of the intervention (and its 95% CI).

CI: confidence interval; **MD:** mean difference; **OR:** odds ratio

GRADE Working Group grades of evidence
High certainty: we are very confident that the true effect lies close to that of the estimate of the effect.
Moderate certainty: we are moderately confident in the effect estimate: the true effect is likely to be close to the estimate of the effect, but there is a possibility that it is substantially different.
Low certainty: our confidence in the effect estimate is limited: the true effect may be substantially different from the estimate of the effect.
Very low certainty: we have very little confidence in the effect estimate: the true effect is likely to be substantially different from the estimate of effect.

Explanations

a. Imprecision was deemed serious due to the width of the CIs, inclusion of the line of no effect or uncertainty over clinical significance of effect.

b. Although there is a suspicion of publication bias, the small number of studies (fewer than 9) limits the reliability of the funnel plot and Egger test in detecting such bias. This limitation makes it challenging to conclusively determine the presence of publication bias, but the potential for bias cannot be ruled out, which may affect the confidence in the estimated effect size.

c. Risk of bias was deemed Serious as the majority of trials had high risk of bias in at least one domain

Funnel Plots for Detecting Publication Bias (Meta-Analyses with >10 Studies)

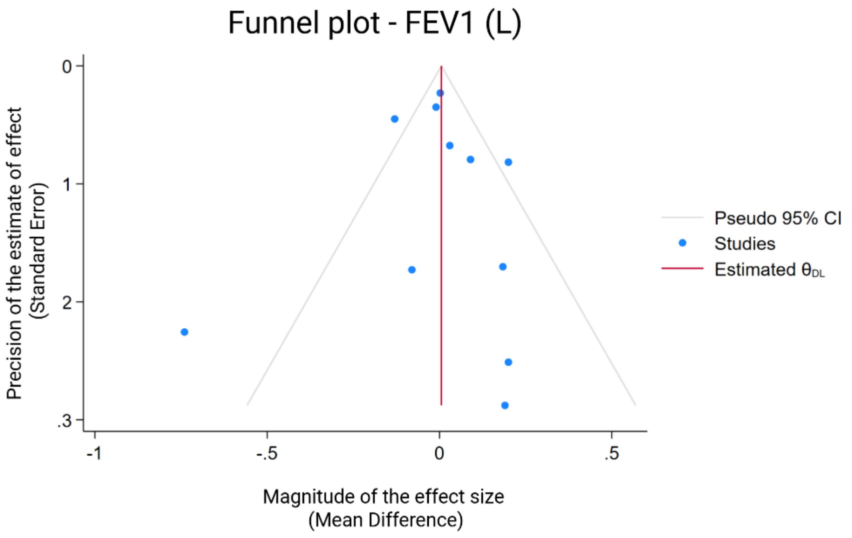

Egger test: p=0.91

Figure S13 – Funnel plot of mean differences in FEV$_1$ (L) between mucoactive treatments and control groups from 11 studies. Egger test used to assess potential publication bias by examining funnel plot asymmetry.

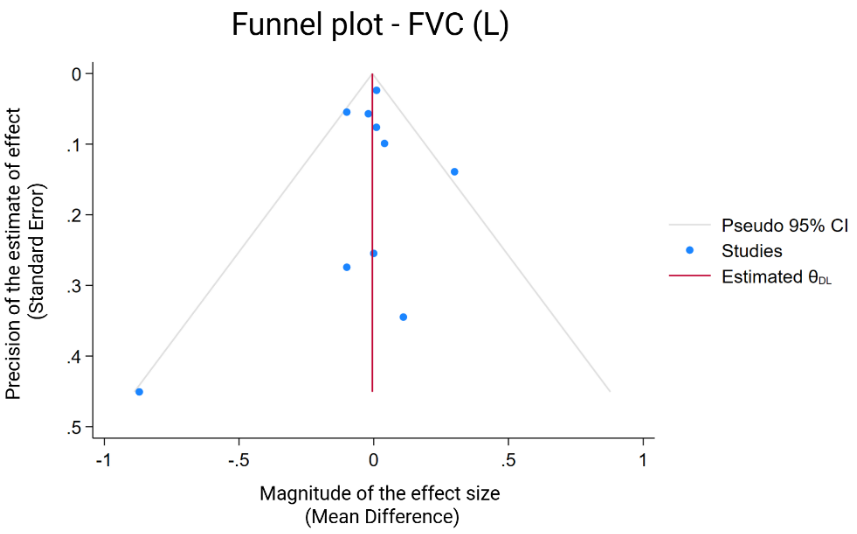

Egger test: p=0.800

Figure S14 – Funnel plot of mean differences in FVC (L) between mucoactive treatments and control groups from ten studies. Egger test used to assess potential publication bias by examining funnel plot asymmetry.
